# Linear Wavelet-Based Estimators of Partial Derivatives of Multivariate Density Function for Stationary and Ergodic Continuous Time Processes

**DOI:** 10.3390/e27040389

**Published:** 2025-04-06

**Authors:** Sultana Didi, Salim Bouzebda

**Affiliations:** 1 Department of Statistics and Operations Research, College of Sciences, Qassim University, P.O. Box 6688, Buraydah 51452, Saudi Arabia; s.biha@qu.edu.sa; 2 LMAC (Laboratory of Applied Mathematics of Compiègne), Université de Technologie de Compiègne, CS 60 319-60 203 Compiègne Cedex, 60203 Compiègne, France

**Keywords:** density estimation, stationarity, ergodicity, rates of strong convergence, wavelet-based estimators, martingale differences, discrete time, stochastic processes, time series, 62G07, 62G08, 62G05, 62G20, 62H05, 60G42, 60G46

## Abstract

In this work, we propose a wavelet-based framework for estimating the derivatives of a density function in the setting of continuous, stationary, and ergodic processes. Our primary focus is the derivation of the integrated mean square error (IMSE) over compact subsets of $R^{d}$, which provides a quantitative measure of the estimation accuracy. In addition, a uniform convergence rate and normality are established. To establish the asymptotic behavior of the proposed estimators, we adopt a martingale approach that accommodates the ergodic nature of the underlying processes. Importantly, beyond ergodicity, our analysis does not require additional assumptions regarding the data. By demonstrating that the wavelet methodology remains valid under these weaker dependence conditions, we extend earlier results originally developed in the context of independent observations.

## 1. Introduction

Multivariate data analysis frequently relies on the behavior of the partial derivatives of underlying density functions, especially for tasks such as identifying modal regions. Despite their significance, research on the nonparametric estimation of higher-order density derivatives remains relatively scarce. The primary goal of this work is to introduce wavelet-based approaches for estimating the partial derivatives of multivariate densities in a nonparametric framework. Estimating the derivatives of unknown functions—whether they are densities or regression functions—constitutes a fundamental statistical operation that underpins numerous applications. Such estimation tasks are common in fields like economics and industrial engineering, where complex systems must be modeled in the face of substantial prior uncertainty. For instance, the second-order derivatives of densities can be used to construct statistical tests for detecting modes [1,2], and they also inform the choice of the bandwidth in kernel density estimation [3]. In nonparametric signal estimation, the ratio between a density’s derivative and the density itself (i.e., the logarithmic derivative) serves a central role in formulating optimal filtering and interpolation equations [4,5]. The accurate estimation of this ratio thereby becomes critical for signal processing. Meanwhile, gradient estimation is essential for isolating filaments in point clouds, a technique frequently employed in medical imaging, remote sensing, seismology, and cosmology [6]. Other motivations include challenges in regression analysis, Fisher information evaluation, parameter estimation, and hypothesis testing [7]. Foundational studies on density derivative estimation can be found in [8,9,10,11,12,13,14,15,16,17,18,19,20,21,22], among others.

Kernel-based estimators are recognized for their strong performance when the underlying target function (whether a density or a regression function) is defined on an unbounded domain. However, they often exhibit deficiencies near the boundaries of a compact support. Furthermore, traditional kernel-based strategies typically assume a sufficiently high degree of smoothness on the target function, for instance, by requiring at least two continuous derivatives. In the absence of such smoothness guarantees, wavelet-based estimators present a compelling alternative. They leverage function spaces that capture refined notions of regularity rather than mere differentiability requirements. Remarkably, although wavelet estimators do not explicitly demand these smoothness properties, they nevertheless attain optimal convergence rates equivalent to those of strategies presupposing full knowledge of the function’s true regularity; see [23].

Wavelet-based methods have thus gained prominence in statistical estimation by deftly accommodating heterogeneous regularity properties and managing discontinuities with ease. An additional merit is their typically low computational overhead, coupled with modest memory usage. For a thorough exposition of wavelet methods in nonparametric function estimation, see [24]. Representative applications of wavelets include the estimation of the integrated squared derivative of a univariate density under independence [25] and under certain forms of dependence [26]. Extensions to the partial derivatives of multivariate densities under independence were explored by [27], while the mixing hypothesis was investigated in [28,29]. More recently, [30] studied wavelet estimators for the partial derivatives of multivariate densities in the presence of additive noise.

Although mixing assumptions are widely employed for their technical convenience, they may not hold when the data exhibit more intricate dependence patterns. In essence, “mixing” conditions entail a form of asymptotic independence that may be unrealistic in settings characterized by strong dependence. Methods that address more general dependence structures remain relatively undeveloped. In this respect, ergodicity provides a broader alternative to strong mixing assumptions by dispensing with their stringent requirements and associated probabilistic complexity [31]. All mixing conditions imply ergodicity; however, ergodicity itself is strictly weaker, enlarging the range of processes that can be analyzed [32]. These advantages are examined in more detail in [33,34], where discussions of ergodic processes and their practical significance can be found. Several examples illustrate that certain processes are ergodic but not mixing [32,35]. In particular, ref. [36] (Section 5.3) constructed a strictly stationary process, ${\left\{\left(T_{i},\lambda_{i}\right)\right\}}_{i\in Z}$, satisfying $T_{i}|T_{i-1}∼\mathrm{Poisson}\left(\lambda_{i}\right)$, with $\lambda_{i}=\kappa \left(\lambda_{i-1},T_{i-1}\right)$ for some mapping, κ. While this process is ergodic under general conditions, it need not be mixing [37]. Another important example involves fractional Brownian motion, $\left\{W_{t}^{H}:t\geq 0\right\}$. When H∈(0,1), this process has strictly stationary increments, and the associated fractional Gaussian noise process $\left\{G_{t}^{H}:t\geq 0\right\}$ exhibits long memory for H>1/2, thereby violating strong mixing [38,39], although suitable correlation decay conditions can still yield ergodicity [40]. In general, verifying ergodicity is often simpler than proving mixing, making ergodic frameworks appealing for analyzing diverse classes of processes, including those arising in chaotic systems with noise [41]. Formally, let ${\left\{X_{n}\right\}}_{n\in Z}$ be a stationary sequence with backward σ-fields, $A_{n}=\sigma \left(X_{k}:k\leq n\right)$, and forward σ-fields, $B_{m}=\sigma \left(X_{k}:k\geq m\right)$. The sequence is deemed *strongly mixing* if

$$\underset{A\in A_{0},\;B\in B_{n}}{\sup}\left|P(A\cap B)-P(A)\;P(B)\right|\;⟶\;0\;\mathrm{as}\;n\to \infty .$$

It is said to be *ergodic* if

$$\frac{1}{n}\sum_{k=0}^{n-1}\left|P\left(A\cap \tau^{-k}B\right)-P\left(A\right)\;P\left(B\right)\right|\;⟶\;0\;\mathrm{as}\;n\to \infty ,$$

where τ denotes the shift operator. Strong mixing implies ergodicity, but the converse does not always hold [32]. Many authors advocate for an ergodic perspective precisely because it accommodates an expanded class of stochastic processes [33,34].

Despite the literature on wavelet estimators for the partial derivatives of multivariate densities, it appears that no existing work addresses dependence structures strictly beyond strong mixing. The present study aims to fill this gap by developing wavelet-based estimators that operate under ergodic conditions. This extension necessitates a suite of martingale techniques that diverge substantially from the standard tools typically employed for strong mixing processes. More importantly, bridging this methodological gap requires novel theoretical contributions specifically tailored to estimating partial derivatives in an ergodic context, rather than a mere combination of pre-existing results obtained using wavelet methods and ergodic theory.

The organization of this paper is as follows. Section 2 is devoted to the mathematical background of the study. The proposed linear wavelet estimators are stated as well. The assumptions and main results concerning the uniform convergence rates and asymptotic normality under the weak dependence assumptions are given in Section 3. Some concluding remarks and suggestions for future work are given in Section 6. Finally, to avoid interrupting the flow of the presentation, the proofs are postponed to Section 7.

### Notation

Throughout the paper, we let *C* be some positive constant, which may be different from one term to another. Set $1_{A}\left(\cdot \right)$ is the indicator function of *A*. For the numerical sequences of positive constants, $a_{n}$, $b_{n}$, where $\left\{a_{n},n\geq 1\right\}$ and $\left\{b_{n},n\geq 1\right\}$, we denote $a_{n}=O\left(b_{n}\right)$ for $a_{n}\leq Cb_{n}$, and $a_{n}=o\left(b_{n}\right)$ implies that $\frac{a_{n}}{b_{n}}\to 0$ as n→∞.

## 2. Mathematical Background

### 2.1. Linear Wavelet Estimator

We begin by reviewing fundamental concepts in wavelet analysis, adopting the notation in [42]. Consider a multiresolution analysis (MRA), ${\left\{V_{j}\right\}}_{j=1}^{\infty }$, of $L_{2}\left(R^{d}\right)$, with ϕ(·) and ψ(·) denoting the scaling function and the orthogonal wavelet, respectively. Both ϕ and ψ are assumed to be compactly supported on ${\left[-L,L\right]}^{d}$ for some L>0 and *r*-regular (for r≥1) if $\phi \left(\cdot \right)\in C^{r}$ and all its partial derivatives up to a total order of *r* are rapidly decreasing, i.e., for every integer i>0, there exists a constant $A_{i}$ such that

(1) $$\left|\left(D^{\beta }\phi \right)\left(x\right)\right|\leq \frac{2^{j(d+|\beta |)/2}A_{i}}{{\left(1+∥x∥\right)}^{i}},\;\mathrm{for}\;\mathrm{all}\;|\beta |\leq r.$$

For every integer *j* and $k\in Z^{d}$, the family

$${\left\{\phi_{j,k}\left(x\right)=2^{\frac{jd}{2}}\;\phi \left(2^{j}x-k\right)\right\}}_{k\in Z^{d}}$$

constitutes an orthonormal basis of $V_{j}$. Furthermore, one obtains $2^{d}-1$ orthonormal wavelet functions:

$$\left\{\psi_{i,j,k}\left(x\right)=2^{\frac{jd}{2}}\;\psi_{i}\left(2^{j}x-k\right);\;i=1,\ldots ,2^{d}-1,\;k\in Z^{d}\right\},$$

which together form an orthonormal basis of $L_{2}\left(R^{d}\right)$.

Next, let ${\left\{X_{i}\right\}}_{i=1}^{n}$ be a collection of (weakly dependent) random variables drawn from a distribution with an unknown density, f(·). Assume *f* is partially differentiable up to a total order of *r*. Our main interest is to estimate the partial derivatives of *f*; that is,

$$\left(\partial^{\beta }f\right)\left(x\right)\;=\;\frac{\partial^{\beta }\;f\left(x\right)}{\partial x_{1}^{\beta_{1}}\;\ldots \;\partial x_{d}^{\beta_{d}}},$$

where $\beta =(\beta_{1},\ldots ,\beta_{d})$ and $\left|\beta \right|=\sum_{i=1}^{d}\beta_{i}$. In particular, we aim to construct wavelet-based estimators of $\left(\partial^{\beta }f\right)\left(\cdot \right)$ that exhibit both strong convergence and asymptotic normality. Assuming that $\left(\partial^{\beta }f\right)\left(\cdot \right)$ lies in $L_{2}\left(R^{d}\right)$, one can invoke [43] to write, for any integer *m*,

$$\left(\partial^{\beta }f\right)\left(x\right)\;=\;\sum_{k\in Z^{d}}\;a_{m,k}\;\phi_{m,k}\left(x\right),$$

where each wavelet coefficient satisfies

$$a_{m,k}\;=\;\int_{R^{d}}\;\left(\partial^{\beta }f\right)\left(u\right)\;\phi_{m,k}\left(u\right)\;du\;=\;{\left(-1\right)}^{\left|\beta \right|}\int_{R^{d}}f\left(u\right)\;\left(\partial^{\beta }\phi_{m,k}\right)\left(u\right)\;du.$$

Here, $\left(\partial^{\beta }\phi_{m,k}\right)\left(\cdot \right)$ denotes the βth partial derivative of $\phi_{m,k}\left(\cdot \right)$.

A natural linear estimator of $\left(\partial^{\beta }f\right)\left(\cdot \right)$ at a resolution level of m=m(n)→∞ is

$${\hat{\left(\partial^{\beta }f\right)}}_{n}\left(x\right)\;=\;\sum_{k\in Z^{d}}\;{\hat{a}}_{m,k}\;\phi_{m,k}\left(x\right),$$

where ${\hat{a}}_{m,k}$ is the following unbiased empirical estimator of $a_{m,k}$:

$${\hat{a}}_{m,k}\;=\;\frac{{\left(-1\right)}^{\left|\beta \right|}}{n}\;\sum_{i=1}^{n}\;\left(\partial^{\beta }\phi_{m,k}\right)\left(X_{i}\right).$$

### 2.2. Besov Spaces

In what follows, we will work within the framework of Besov spaces, $B_{s,p,q}$ (with s>0, 1≤p≤∞, and 1≤q≤∞). According to [42], these spaces can be characterized using wavelet coefficients. Specifically, if 0<s<r and $f\left(\cdot \right)\in L_{p}\left(R^{d}\right)$ (where *s* captures the real-valued smoothness of *f*), then the membership $f\left(\cdot \right)\in B_{s,p,q}$ is equivalent to the following two conditions:

(B.1) $$J_{s,p,q}\left(f\right)\;=\;{∥P_{V_{0}}f∥}_{L_{p}}\;+\;{\left(\sum_{j>0}{\left(2^{js}\;{∥P_{W_{j}}f∥}_{L_{p}}\right)}^{q}\right)}^{1/q}\;<\;\infty ,$$
(B.2) $$J_{s,p,q}^{\prime }\left(f\right)\;=\;{∥a_{0,\cdot }∥}_{l_{p}}\;+\;{\left(\sum_{j>0}{\left(2^{\;j\;[s\;+\;d\left(\frac{1}{2}-\frac{1}{p}\right)]}\;{∥b_{j,\cdot }∥}_{l_{p}}\right)}^{q}\right)}^{1/q}\;<\;\infty ,$$
  where
  $$a_{0,\cdot }\;=\;\int_{R^{d}}f\left(u\right)\;\phi_{0,k}\left(u\right)\;du,\;b_{i,j,k}\;=\;\int_{R^{d}}f\left(u\right)\;\psi_{i,j,k}\left(u\right)\;du,$$
  and
  $${∥b_{j,\cdot }∥}_{l_{p}}\;=\;{\left(\sum_{i=1}^{d}\sum_{k\in Z^{d}}{\left|b_{i,j,k}\right|}^{p}\right)}^{1/p}.$$
  When q=∞, one uses the corresponding supremum norm. Notably, these Besov spaces encapsulate various function classes frequently used in statistical analysis, including the Hilbert space $H^{s}=B_{s,2,2}$ and the space of bounded *s*-Lipschitz functions, $C^{s}=B_{s,\infty ,\infty }$ (for a non-integer *s*), among others. Besov spaces, $B_{s,p,q}$, are of central importance in statistical estimation and approximation theory, as they provide a flexible framework for characterizing smoothness properties; for instance, see [44,45,46,47,48]. Additional equivalent characterizations and advantages of Besov spaces in approximation theory and statistics can be found in [49,50,51,52] and in the Appendix A.

### 2.3. Linear Wavelet Estimator

We begin by reviewing key elements of wavelet theory, following the notation introduced in [42]. Consider a multiresolution analysis, ${\left\{V_{j}\right\}}_{j=1}^{\infty }$, of the space $L_{2}\left(R^{d}\right)$. Let ϕ(·) be the scaling function and ψ(·) the orthogonal wavelet function, both assumed to be *r*-regular (with r≥1) and compactly supported within ${\left[-L,L\right]}^{d}$ for some L>0. For each integer *j* and each $k\in Z^{d}$, define

$$\phi_{j,k}\left(x\right)\;=\;2^{jd/2}\;\phi \left(2^{j}x\;-\;k\right).$$

It is well known that ${\left\{\phi_{j,k}\right\}}_{k\in Z^{d}}$ forms an orthonormal basis for $V_{j}$. Furthermore, there exist $2^{d}-1$ associated wavelet functions, giving rise to the family

$$\left\{\psi_{i,j,k}\left(x\right)\;=\;2^{jd/2}\;\psi_{i}\left(2^{j}x-k\right)\;|\;i=1,\ldots ,2^{d}-1,\;k\in Z^{d}\right\},$$

which constitutes an orthonormal basis for $L_{2}\left(R^{d}\right)$.

Let ${\left\{X_{i}\right\}}_{i\geq 1}$ be an $R^{d}$-valued strictly stationary ergodic process defined on a probability space, (Ω,A,P). Let *f* be the common density function of the sample $X_{1},\ldots ,X_{n}$, which is assumed to be bounded and continuous, that is partially differentiable up to a total order of *r*. In this work, our principal objective is to estimate the partial derivative of the density:

$$\left(\partial^{\beta }f\right)\left(x\right)\;=\;\frac{\partial^{\beta }f\left(x\right)}{\partial x_{1}^{\beta_{1}}\;\ldots \;\partial x_{d}^{\beta_{d}}},$$

where $\beta =(\beta_{1},\ldots ,\beta_{d})$ and $\left|\beta \right|=\sum_{i=1}^{d}\beta_{i}$. As highlighted in the Introduction, we aim to construct wavelet-based estimators of $\left(\partial^{\beta }f\right)\left(\cdot \right)$ that exhibit both MISE and strong convergence as well as asymptotic normality.

According to [43], for any integer *m*, this derivative of the density can be expanded in the subspace $V_{m}$ as

$$\left(\partial^{\beta }f\right)\left(x\right)\;=\;\sum_{k\in Z^{d}}a_{m,k}\;\phi_{m,k}\left(x\right),$$

where each wavelet coefficient, $a_{m,k}$, is given by

$$a_{m,k}\;=\;\int_{R^{d}}\left(\partial^{\beta }f\right)\left(u\right)\;\phi_{m,k}\left(u\right)\;du\;=\;{\left(-1\right)}^{\left|\beta \right|}\int_{R^{d}}f\left(u\right)\;\left(\partial^{\beta }\phi_{m,k}\right)\left(u\right)\;du,$$

where we have applied multiple integration by parts.

**Remark 1.**  *The fact that ϕ(·) and $\psi_{i}\left(\cdot \right)$ are bounded and compactly supported, with the support being the monotonically increasing function of their degree of differentiability, ensures that the above summations on $k\in Z^{d}$ are finite for each fixed x (i.e., they converge in a pointwise sense; for instance, see [43]).*

Note that $\left(\partial^{\beta }\phi_{m,k}\right)\left(\cdot \right)$ denotes the βth partial derivative of $\phi_{m,k}\left(\cdot \right)$. We define a linear estimator of $\left(\partial^{\beta }f\right)\left(\cdot \right)$ at the resolution level m=m(n)→∞ (to be specified later) by

(2) $${\hat{\left(\partial^{\beta }f\right)}}_{T}\left(x\right)\;=\;\sum_{k\in Z^{d}}{\hat{a}}_{m,k}\;\phi_{m,k}\left(x\right),$$

where the unbiased empirical estimate ${\hat{a}}_{m,k}$ of $a_{m,k}$ is given by

(3) $${\hat{a}}_{m,k}\;=\;\frac{{\left(-1\right)}^{\left|\beta \right|}}{T}\int_{0}^{T}\left(\partial^{\beta }\phi_{m,k}\right)\left(X_{t}\right)dt.$$

This construction will serve as the foundation for our subsequent analysis.

## 3. Assumptions and Main Results

We introduce the following notation and our hypotheses to facilitate the presentation of our main results. For the remainder of the paper, any small, real 0≤t≤T and δ>0, we will denote by

$$n=\frac{T}{\delta }\in N,\;and\;T_{j}=j\delta ,\;for\;j=1,\ldots ,n.$$

Let $F_{t-\delta }$ be the σ-field generated by

$$\left\{(X_{s}):0\leq s<t-\delta \right\}.$$

Let $F_{j}$ be the σ-field generated by

$$\left\{\left(X_{s}\right):0\leq s\leq T_{j}\right\}.$$

We define the conditional density of $X_{i}$ given $F_{i-1}$ as

$$f_{X_{t}}^{F_{t-\delta }}\left(\cdot \right)\;=\;f_{t}^{F_{t-\delta }}\left(\cdot \right).$$

The following assumptions are imposed throughout the paper.

**(C.1)**  For every x∈S, the sequence
  $$\frac{1}{T}\int_{0}^{T}f_{t}^{F_{t-\delta }}\left(x\right)dt,$$
  converges to f(x) as n→∞, both almost surely (a.s.) and in the $L^{2}$ sense.
**(C.2)**  Moreover, for every x∈S,
  $$\lim_{n\to \infty }\underset{x\in R^{d}}{\sup}\left|\frac{1}{T}\int_{0}^{T}f_{t}^{F_{t-\delta }}\left(x\right)dt\;-\;f\left(x\right)\right|\;=\;0,$$
  again in both the almost sure and $L^{2}$ senses.
**(C.3)**  The partial derivative function $\left(\partial^{\beta }f\right)\left(\cdot \right)$ belongs to the Besov space $B_{s,p,q}$ for some 0<s<r, l≤p,q≤∞.
**(C.4)**  For all values of i=1;…;n, the partial derivative function $\left(\partial^{\beta }f^{F_{i-1}}\right)\left(\cdot \right)$ belongs to the Besov space $B_{s,p,q}$ for some 0<s<r, l≤p,q≤∞.

Define the kernel K(u,v) by

(4) $$K\left(u,v\right):=\sum_{k\in Z^{d}}\phi \left(u-k\right)\phi \left(v-k\right)\;\;and\;\;h_{n}=2^{-m(n)}.$$

Consequently,

(5) $$K^{\left(\beta \right)}\left(u,v\right):=\sum_{k\in Z^{d}}\phi \left(u-k\right)\left(\partial_{v}^{\beta }\phi \right)\left(v-k\right),$$

where $K^{\left(\beta \right)}\left(u,v\right):=\left(\partial_{v}^{\beta }k\right)\left(u,v\right)$ denotes the βth partial derivative of K(u,v) with respect to v. We infer that the derivative kernel function $K^{\left(\beta \right)}\left(\cdot \right)$ defined in (5) converges uniformly and satisfies [42] (p. 33), i.e., for ∣α∣≤r, ∣β∣≤r, and for some constant $C_{m}>0$ with m≥1, the following inequality holds:

$$|\left(\partial_{u}^{\alpha }\partial_{v}^{\beta }K\right)\left(u,v\right)|\leq \frac{C_{m}}{{(1+∥v-u∥}_{2}{)}^{m}},$$

where, for any $x=\left(x_{1},\ldots ,x_{d}\right)\in \in R^{d},{∥x∥}_{2}=\sqrt{\sum_{i=1}^{d}x_{i}^{2}}$. For α=0 and m=d+|β|, we obtain

(6) $$|K^{\left(\beta \right)}\left(u,v\right)|\leq \frac{C_{d+|\beta |}}{{(1+∥v-u∥}_{2}{)}^{d+|\beta |}}.$$

That is, for any j≥1, we have

$$\int_{R^{d}}{\left|K^{\left(\beta \right)}\left(v,u\right)\right|}^{j}dv\leq G_{j}\left(d\right).$$

The function $G_{j}\left(d\right)$ is defined as

$$G_{j}\left(d\right)=2\pi^{d/2}\frac{\Gamma (d)\Gamma (j+d(j-1))}{\Gamma (d/2)\Gamma ((d+1)j)}C_{d+1}^{j},$$

where Γ(t) represents the Gamma function, given by

$$\Gamma \left(t\right):=\int_{0}^{\infty }y^{t-1}e^{-y}\;dy.$$

Furthermore, by assuming |α|=1 and m=2, we deduce that

(7) $$\left|\frac{\partial_{u}\partial_{v}^{\beta }K\left(u,y\right)}{\partial u_{i}}\right|\leq \frac{C_{m}}{{(1+∥u-y∥}_{2}{)}^{2}}\leq C_{2},\;i=1,\ldots ,d.$$

This, in turn, implies that

(8) $$\left|K^{\beta }\left(u,y\right)-K^{\beta }\left(v,y\right)\right|\leq C_{2}\sum_{i=1}^{d}|u_{i}-v_{i}|\leq d^{1/2}C_{2}{∥u-v∥}_{2}.$$

By incorporating Equations (2), (3), and (5), the estimator ${\hat{\left(\partial^{\beta }f\right)}}_{n}\left(x\right)$ can be expressed in an extended kernel estimation framework as follows:

(9) $${\hat{\left(\partial^{\beta }f\right)}}_{T}\left(x\right)=\frac{{\left(-1\right)}^{\left|\beta \right|}}{Th_{T}^{d+|\beta |}}\int_{0}^{T}K^{\left(\beta \right)}\left(\frac{x}{h_{T}},\frac{X_{t}}{h_{T}}\right)dt,\;\mathrm{where}\;h_{T}=2^{-m(T)}.$$

We refer the reader to [27] for further details.

## 4. Comments on Hypotheses

Assume that the random functions, $f_{t}^{F_{t-\delta }}\left(x\right)$, for any t∈[0,T], belong to the space, $C^{0}$, of continuous functions, which is a separable Banach space. Moreover, approaching the integral $\int_{0}^{T}f_{t}^{G_{t-\delta }}\left(x\right)dt$ through its Riemann’s sum, it follows that

$$\begin{array}{ccc} \frac{1}{T}\int_{0}^{T}f_{t}^{F_{t-\delta }}\left(x\right)dt & = & \frac{1}{T}\sum_{i=1}^{n}\int_{T_{i-1}}^{T_{i}}f_{t}^{F_{t-\delta }}\left(x\right)dt\\ & ⋍ & \frac{1}{n}\sum_{i=1}^{n}f_{T_{i-1}}^{F_{T_{i-2}}}\left(x\right). \end{array}$$

Since the process ${\left(X_{T_{j}}\right)}_{j\geq 1}$ is stationary and ergodic, following [53] (see Lemma 4 and Corollary 1, together with their proofs), one may prove that the sequence, ${\left(f_{j\delta ,(j-1)\delta }\left(x\right)\right)}_{j\geq 1}$, of random functions is stationary and ergodic. Indeed, in the work of [53], it sufficed to replace the conditional densities with $f_{j\delta ,(j-1)\delta }$ and the density with the function f(·). In nonparametric estimation, boundedness conditions (C.3) and (C.4) are typically assumed [54]. These conditions can be relaxed by using weighted approximations or distances [55,56]. However, this relaxation requires developing new probabilistic tools for the empirical process, tools that have not yet been established for dependent cases.

**Theorem 1.**  *Under the stated assumptions (C.1) and (C.3), let f be an element of the Besov space $B_{s,p,q}$ with s>1/p and 1≤p,q≤∞. In this setting, the linear wavelet estimator ${\hat{\left(\partial^{\beta }f\right)}}_{n}$ satisfies*

$$E{∥{\hat{\left(\partial^{\beta }f\right)}}_{T}-\partial^{\beta }f∥}_{2}^{2}\;=\;O\;(T^{-\frac{2(s-|\beta |)}{2s+1}}).$$

**Theorem 2.**  *Assume that*

$$m\left(T\right)=m\to \infty \;\;and\;\;\frac{2^{dm(T)}\log T}{T}\to 0\;\;as\;\;n\to \infty .$$

*For every compact subset, $D\subset R^{d}$, under assumptions (C.1) and (C.3), we have, almost surely,*

$$\underset{x\in D}{\sup}\left|{\hat{\left(\partial^{\beta }f\right)}}_{T}\left(x\right)-E\left[{\hat{\left(\partial^{\beta }f\right)}}_{T}\left(x\right)\right]\right|=O\left({\left(\frac{\log T}{Th_{T}^{d+2|\beta |}}\right)}^{1/2}\right)+O\left(h_{T}^{-1/2}\right).$$

A result of the bias term is given in the following lemma.

**Lemma 1.**  *Using the assumption (C.4), we obtain the following result:*

$$B_{T}\left(x\right)={∥E\left[{\hat{\left(\partial^{\beta }f\right)}}_{T}\left(x\right)\right]-\left(\partial^{\beta }f\right)\left(x\right)∥}_{L^{\infty }}=O\left(h_{T}^{\delta }\right),$$

*for $\delta =s-\frac{d}{p}>0$.*

**Remark 2.**  *The term $2^{-m(T)}$ in the preceding theorems is directly linked to the bandwidth $h_{T}$ used in Parzen–Rosenblatt kernel density estimation. In practical applications, determining the multiresolution level m(T) in the wavelet framework tends to be more straightforward than selecting the bandwidth $h_{T}$. This is because, in practice, only a limited set of values for m(T) (typically three or four) needs to be considered, making the procedure both more intuitive and computationally efficient.*

**Remark 3.**  *It is well established that kernel estimators lose accuracy as the dimensionality of the data increases. This phenomenon, widely known as the* curse of dimensionality* [57], arises because in high-dimensional settings, local neighborhoods require remarkably large sample sizes to gather an adequate number of observations. Consequently, unless the sample size is extremely large, one must use bandwidths so wide that the concept of truly “local” averaging is essentially lost. A detailed account of these challenges, including numerical examples, can be found in [58], while more recent analyses appear in [59,60]. Notwithstanding the popularity of penalized splines, considerable uncertainty persists regarding their asymptotic performance even in conventional nonparametric problems, and only a handful of theoretical studies have examined their behavior. Furthermore, most functional regression methods rely on minimizing an $L_{2}$ norm and thus remain susceptible to outliers. Alternative, less conventional but potentially effective strategies include approaches employing delta sequences and wavelet-based techniques [61].*

**Remark 4.**  *An intriguing avenue for future research lies in broadening the current theoretical framework to accommodate processes that exhibit memory, including non-Markovian stochastic dynamics. However, realizing such an extension will likely necessitate a methodological departure from the techniques employed here. Consequently, we leave the pursuit of this challenging direction open for subsequent investigations.*

### 4.1. Asymptotic Normality Results

In this section, we establish a central limit theorem for the estimator defined in Equation (2) under the assumption of weak dependence. Our findings are derived under straightforward regularity conditions on the estimator and minimal bandwidth requirements. Notably, the mathematical premises and outcomes of Theorem 1 remain applicable in subsequent discussions.

We denote by $Z_{n}\overset{D}{\to }N\left(0,\Sigma^{2}\left(x\right)\right)$ that the sequence of random variables ${\left(Z_{n}\right)}_{n\geq 1}$ converges in distribution to a normal distribution with a mean of zero and the covariance matrix $\Sigma^{2}\left(x\right)$.

**Theorem 3.**  *Assume that the hypotheses (C.1)–(C.4) are satisfied. Additionally, suppose that*

(10) $$Th_{T}^{d+2|\beta |+2\delta }\to 0\;as\;T\to \infty .\;Th_{T}^{2d+2|\beta |}\to 0\;as\;T\to \infty .$$

*Then, the following convergence holds:*

$$\sqrt{Th_{T}^{d+2|\beta |}}\left({\hat{\left(\partial^{\beta }f\right)}}_{T}\left(x\right)-\left(\partial^{\beta }f\right)\left(x\right)\right)\overset{D}{\to }N\left(0,\Sigma^{2}\left(x\right)\right),$$

*where*

$$\Sigma^{2}\left(x\right):=\Sigma_{\left(\beta \right)}^{2}\left(x\right):=f_{X}\left(x\right)\int_{R^{d}}{\left(K^{\left(\beta \right)}\right)}^{2}\left(0,u\right)\;du.$$

The proof of Theorem 3 is detailed in Section 7.

**Remark 5.**  *By leveraging Theorem 1, we can derive a novel result regarding the MSE of the wavelet-based multivariate density estimator. Specifically, under suitable conditions, we establish that*

$$E{∥{\hat{f}}_{T}-f∥}_{2}^{2}\;=\;O\;(T^{-\frac{2s}{2s+1}}).$$

*Furthermore, employing a similar analytical framework to Theorem 3, we obtain an asymptotic normality result. In particular, we show that*

$$\sqrt{\;T\;h_{T}^{d}}\;\left({\hat{f}}_{T}\left(x\right)\;-\;f\left(x\right)\right)\;\;\overset{D}{⟶}\;\;N\;\left(0,\Sigma_{\left(0\right)}^{2}\left(x\right)\right),$$

*where the asymptotic variance is given by*

$$\Sigma_{\left(0\right)}^{2}\left(x\right)\;=\;f_{X}\left(x\right)\int_{R^{d}}K^{2}\left(0,u\right)\;du.$$

*These results align with the theoretical insights presented in Theorem 4.7 of [33], reinforcing the effectiveness of wavelet estimators in nonparametric multivariate settings.*

### 4.2. Confidence Interval

The asymptotic variance $\Sigma^{2}\left(x\right)$ in the central limit theorem depends on the unknown density function $f_{X}\left(\cdot \right)$ of X and must be estimated for practical applications. This estimation involves selecting a suitable family of bounded, compactly supported discrete wavelets from the existing literature (such as the commonly utilized Daubechies wavelets [43]) with an adequately large multiresolution level, m(T), and an adaptive initial level, $j_{0}$. The unknown parameters are chosen using wavelet-based methods combined with a plug-in approach. We define ${\hat{\Sigma }}^{2}\left(x\right)$ as a consistent estimator of the variance $\Sigma^{2}\left(x\right)$ by setting

$${\hat{\Sigma }}^{2}\left(x\right):={\hat{f}}_{X}\left(x\right)\int_{R^{d}}{\left(K^{\left(\beta \right)}\right)}^{2}\left(0,u\right)\;du,$$

where ${\hat{f}}_{X}\left(\cdot \right)$ is a consistent estimator of $f_{X}\left(\cdot \right)$. Consequently, an approximate confidence interval for $\left(\partial^{\beta }f\right)\left(x\right)$ can be constructed as

$$\left(\partial^{\beta }f\right)\left(x\right)\in \left[{\hat{\left(\partial^{\beta }f\right)}}_{T}\left(x\right)\pm c_{\alpha }\frac{{\hat{\Sigma }}^{2}\left(x\right)}{\sqrt{Th_{T}^{d+2\beta }}}\right],$$

where $c_{\alpha }$ represents the (1−α) quantile of the standard normal distribution.

## 5. Application to Multivariate Mode Estimation

In this section, we address the problem of estimating the nonparametric mode, following the framework established in [62] and retaining the same notation and definitions. The kernel mode estimator is defined as any random variable, ${\hat{\Theta }}_{T}$, that satisfies

(11) $${\hat{f}}_{T}\left({\hat{\Theta }}_{T}\right)=\underset{x\in R^{p}}{\sup}{\hat{f}}_{T}\left(x\right),$$

where ${\hat{\left(\partial^{0}f\right)}}_{T}={\hat{f}}_{T}$. This estimator can be explicitly characterized as

$${\hat{\Theta }}_{T}=\inf\left\{y\in R^{d}|{\hat{f}}_{T}\left(y\right)=\underset{x\in R^{d}}{\sup}{\hat{f}}_{T}\left(x\right)\right\},$$

where the infimum is taken with respect to the lexicographic order on $R^{p}$. This definition ensures the measurability of the wavelet-based mode estimator. Assuming that the true mode Θ is nondegenerate, meaning that the Hessian matrix $D^{2}f\left(\Theta \right)$ (the second-order derivative at Θ) is nonsingular, we denote by ∇ℓ(·) the gradient of ℓ(·). From the definition of ${\hat{\Theta }}_{T}$, it follows that

$$\nabla {\hat{f}}_{T}\left({\hat{\Theta }}_{T}\right)=0.$$

Rearranging this, we obtain

(12) $$\nabla {\hat{f}}_{T}\left({\hat{\Theta }}_{T}\right)-\nabla {\hat{f}}_{T}\left(\Theta \right)=-\nabla {\hat{f}}_{T}\left(\Theta \right).$$

Applying Taylor’s expansion to the partial derivative $\frac{\partial f_{n}\left(\cdot \right)}{\partial x_{i}}$, we find that there exists a vector, $\xi_{T}\left(i\right)={\left(\xi_{T;1}\left(i\right),\ldots ,\xi_{T;d}\left(i\right)\right)}^{⊤}$, such that

$$\frac{\partial {\hat{f}}_{T}}{\partial x_{i}}\left({\hat{\Theta }}_{T}\right)-\frac{\partial {\hat{f}}_{n}}{\partial x_{i}}\left(\Theta \right)=\sum_{j=1}^{d}\frac{\partial^{2}{\hat{f}}_{T}}{\partial x_{i}\partial x_{j}}\left(\xi_{T}\left(i\right)\right)\left({\hat{\theta }}_{T,j}-\theta_{j}\right),$$

where $|\xi_{T;j}\left(i\right)-\theta_{j}\left|\;\leq \;\right|{\hat{\theta }}_{T,j}-\theta_{j}|$ for all j=1,…,d. Next, define the d×d matrix $H_{T}={\left(H_{T,i,j}\right)}_{1\leq i,j\leq d}$ as

$$H_{T,i,j}=\frac{\partial^{2}{\hat{f}}_{T}}{\partial x_{i}\partial x_{j}}\left(\xi_{T}\left(i\right)\right).$$

Substituting this into (12), we obtain

(13) $$H_{T}\left({\hat{\Theta }}_{T}-\Theta \right)=-\nabla {\hat{f}}_{T}\left(\Theta \right).$$

Applying the first result of Theorem 2, we establish that

$$\lim_{T\to \infty }\underset{x\in R^{d}}{\sup}\left|\frac{\partial^{2}{\hat{f}}_{T}}{\partial x_{i}\partial x_{j}}\left(x\right)-E\left(\frac{\partial^{2}{\hat{f}}_{T}}{\partial x_{i}\partial x_{j}}\left(x\right)\right)\right|=0,\;a.s.$$

Moreover, standard results ensure that $E\left(\frac{\partial^{2}{\hat{f}}_{T}}{\partial x_{i}\partial x_{j}}\left(x\right)\right)$ converges uniformly to $\frac{\partial^{2}f}{\partial x_{i}\partial x_{j}}\left(x\right)$ in a neighborhood of Θ. Consequently, it follows that

$$\lim_{T\to \infty }{\hat{\Theta }}_{T}=\Theta ,\;a.s.$$

which further implies

$$\lim_{T\to \infty }H_{T}=D^{2}f\left(\Theta \right).$$

From Equation (13), the convergence rate of ${\hat{\Theta }}_{T}-\Theta$ is determined using the behavior of $D^{2}f\left(\Theta \right)\nabla f_{T}\left(\Theta \right)$. Under appropriate regularity conditions and leveraging Theorem 2, we obtain

(14) $$\begin{array}{c} \left|{\hat{\Theta }}_{T}-\Theta \right|=O\left({\left(\frac{\log T}{Th_{T}^{d+2|\beta |}}\right)}^{1/2}\right)+O\left(h_{T}^{s-\frac{d}{p}}\right). \end{array}$$

For related results on modal regression, see [41].

**Remark 6.**  *It is well established that the performance of kernel estimators deteriorates as the dimensionality of the data increases. This issue, commonly referred to as the curse of dimensionality [57], arises because, in high-dimensional spaces, a substantial number of observations are required within local regions to ensure reliable estimation. However, unless the sample size is exceptionally large, the bandwidth must be set so broadly that the fundamental principle of “local” averaging is undermined. A comprehensive discussion of these challenges, along with numerical demonstrations, is available in [58], and further insights can be found in more recent studies [59,60]. Although penalized splines remain widely used, their asymptotic properties remain insufficiently understood, even in conventional nonparametric frameworks. Theoretical studies examining their long-term behavior are relatively scarce. Additionally, it is important to recognize that many functional regression methodologies rely on minimizing the $L_{2}$ norm, making them particularly vulnerable to the influence of outliers. Alternative approaches, which may provide more robust solutions, include methods based on delta sequences and wavelet techniques [61].*

**Remark 7.**  *Let us recall some integral functions of the density function*

$$T_{1}\left(F\right)=\int_{R}{\left(f^{\prime }\left(x\right)\right)}^{2}\;dx,\;T_{2}\left(F\right)=-\int_{R}a\left(F\left(x\right)\right)f^{\prime }\left(x\right)dx\;\;and\;\;T_{3}\left(F\right)=\int_{R}{\left(f\left(x\right)\right)}^{2}\;dx.$$

*Notice that the functional $T_{3}\left(F\right)$ is a special case of $T_{2}\left(F\right)$. The functionals $T_{1}\left(F\right)$ and $T_{3}\left(F\right)$ appear in plug-in data-driven bandwidth selection procedures in density estimation (refer to [63] and the references therein), and the functional $T_{2}\left(F\right)$ arises as part of the variance in nonparametric location and regression estimation based on linear rank statistics (refer especially to [64]). Consider the following general class of the integral functionals of the density:*

$$T\left(F\right)=\int_{R}\varphi \left(x,F\left(x\right),F^{\left(1\right)}\left(x\right),\ldots ,F^{\left(r\right)}\left(x\right)\right)dF\left(x\right),$$

*where F is a cumulative distribution function on R with r≥1 derivatives, $F^{\left(m\right)}$; refer to [65,66] for more details. One can estimate T(F) through plug-in methods making use of the wavelet estimate of the density function and its derivatives. The proof of such a statement, however, should require a different methodology than that used in the present paper, and we leave this problem open for future research.*

**Remark 8.**  *The nonlinear thresholding method provides alternative estimators to $f_{X}\left(x\right)$. These estimators are constructed as follows:*

(15) $${\hat{f}}_{X}\left(x\right)=\sum_{k\in Z^{d}}{\hat{a}}_{j_{0},k}\;\phi_{j_{0},k}\left(x\right)\;+\;\sum_{j=j_{0}}^{\tau }\sum_{i=1}^{N}\sum_{k\in Z^{d}}{\hat{b}}_{ijk}\;1\;\left\{\left|{\hat{b}}_{ijk}\right|\;>\;\delta_{j,T}\right\}\;\psi_{i,j,k}\left(x\right),$$

*where $\delta_{j,T}$ represents a carefully chosen threshold. In the case of a univariate setting (d=1), the estimator ${\hat{f}}_{X}$ was originally introduced by [49]. Further investigation into the effectiveness and adaptability of these estimators in higher-dimensional settings would be a promising avenue for future research.*

**Remark 9.**  *The convergence rates in the sup-norm established in the theorems are comparable to those obtained in [23,67,68,69], where optimal estimation results were derived. Notably, the precise logarithmic rates of convergence are influenced by the resolution level, which in turn depends on the smoothness parameter s of the function f(·) defined within the Besov space $B_{s,p,q}$. This characteristic is a well-known feature of nonparametric estimation techniques and is consistent with most references in the literature. The smoothness assumptions considered in this work extend beyond the standard integer-order differentiability conditions typically required for convolution kernel methods, even though the exact value of s is generally unknown.*

*To ensure the estimator is applicable in practice, various techniques have been introduced for selecting the optimal adaptive value of m(T). Among the most widely used methods are “Stein’s method”, the “rule of thumb”, and “cross-validation”. For a comprehensive discussion on these approaches and their role in deriving asymptotically optimal empirical bandwidth selection rules, we refer to [24,70].*

**Remark 10.**  *To provide insight into the validity of our assumptions as outlined in [71], we present several examples that illustrate how these conditions hold in different settings:*

*1*. *Long memory discrete time processes: Consider a white noise sequence, ${\left(\epsilon_{t}\right)}_{t\in Z}$, with the variance $\sigma^{2}$, and let I and B denote the identity and backshift operators, respectively. As established in [72] (Theorem 1, p. 55), the k-factor Gegenbauer process is given by*
$$\prod_{i\leq i\leq k}{\left(I-2v_{i}B+B^{2}\right)}^{d_{i}}X_{t}=\epsilon_{t},$$
  *where the parameters satisfy $0<d_{i}<1/2$ for $|\nu_{i}|<1$ and $0<d_{i}<1/4$ for $|\nu_{i}|=1$, with i=1,…,k. This process is characterized by stationarity, causality, invertibility, and long memory behavior. Additionally, it admits a moving average representation, $X_{t}=\sum_{j\geq 0}\psi_{j}\left(d,v\right)\epsilon_{t-j}$, where the condition $\sum_{j=0}^{\infty }\psi_{j}^{2}\left(d,v\right)<\infty$ ensures its asymptotic stability.*
*However, [73] demonstrated that when ${\left(\epsilon_{t}\right)}_{t\in Z}$ follows a Gaussian distribution, the process is not strongly mixing. Despite this, the moving average formulation guarantees that the process remains stationary, Gaussian, and ergodic. This example underscores the nuanced role of mixing conditions and highlights the significance of the moving average representation in understanding long-term dependencies.*
*2*. *The stationary solution of a linear Markov AR(1) process: Consider the autoregressive process defined by $X_{i}=\frac{1}{2}X_{i-1}+\epsilon_{i}$, where $\left(\epsilon_{i}\right)$ are independent symmetric Bernoulli variables taking the values −1 and 1. As established in [35], this process does not satisfy the α-mixing property due to its intrinsic dependency structure. Nonetheless, it retains key statistical properties such as stationarity, Markovianity, and ergodicity. This example illustrates that a process can be Markovian and ergodic without necessarily being strongly mixing, which has important implications for statistical inference in time series and functional data analysis.*
*3*. *A stationary process with an AR(1) representation: Consider an independent and identically distributed (i.i.d.) sequence, $\left(u_{i}\right)$, uniformly distributed over {1,…,9}, and define the process as*
$$X_{t}:=\sum_{i=0}^{\infty }10^{-i-1}u_{t-i},$$
  *where $u_{t},u_{t-1},\ldots$ represent the decimal expansion of $X_{t}$. This process satisfies stationarity and can be expressed in an AR(1) form:*
$$X_{t}=\frac{1}{10}X_{t-1}+\frac{1}{10}u_{t}=\frac{1}{10}X_{t-1}+\frac{1}{2}+\epsilon_{t},$$
  *where $\epsilon_{t}=\frac{1}{10}u_{t}-\frac{1}{2}$ constitutes a strong white noise process. Although this process does not satisfy the α-mixing property [74] (Example A.3, p. 349), it remains ergodic. This example highlights that even in the absence of strong mixing, ergodicity can be preserved, making the process applicable in areas such as nonparametric functional data analysis.*

**Remark 11.**  *In many continuous time settings, observations are obtained through a sampling design. The literature contains several approaches to discretization, including both deterministic and randomized schemes; see, for instance, [75,76,77,78,79]. To illustrate this, let us consider the estimation of the density function f(·) from the continuous time sample $\left\{X_{t}:t\in \left[0,T\right]\right\}$ by means of discrete observations, $\left\{X\left(t_{k}\right):k=1,\ldots ,n\right\}$. A natural kernel-based density estimator is therefore*

$$f_{n}\left(x\right)\;=\;\frac{1}{n\;h_{n}}\sum_{j=1}^{n}K\;\left(\frac{x\;-\;X_{t_{j}}}{h_{n}^{\;1/p}}\right).$$

*Following [75], we focus on two types of sampling designs: deterministic sampling and random sampling.*

**Deterministic sampling.**  *Suppose the observation times ${\left(t_{k}\right)}_{1\leq k\leq n}$ are irregularly spaced but deterministic and satisfy*
$$\underset{1\leq k\leq n}{\inf}\left|t_{k+1}-t_{k}\right|\;=\;\frac{1}{\tau }$$
  *for some constant τ>0. For each 1≤k≤n, let $G_{k}=\sigma \left(X(t_{k})\right)$ denote the σ-field generated by all observations, $\left\{X_{s}:0\leq s\leq t_{k}\right\}$. Evidently, the sequence ${\left(G_{k}\right)}_{k=1}^{n}$ forms an increasing family of σ-fields.*
**Random sampling.**  *Assume the sampling times ${\left(t_{k}\right)}_{1\leq k\leq n}$ are independent random variables uniformly distributed on [0,T], independent of the process $\left\{X_{t}:t\in \left[0,T\right]\right\}$. Denote by*
$$0\;\leq \;\tau_{1}\;<\;\tau_{2}\;<\;\cdots \;<\;\tau_{n}\;\leq \;T$$
  *the corresponding order statistics, which serve as the actual observation points. Since these times are strictly increasing, their spacings are strictly positive. Defining $G_{k}=\sigma \left(X(t_{k})\right)$ as the σ-field generated by $\left\{X_{s}:0\leq s\leq \tau_{k}\right\}$ again yields an increasing sequence, ${\left(G_{k}\right)}_{k=1}^{n}$.*

*We also note that a penalization method for selecting the mesh δ of observations achieves the optimal rate of convergence. However, we leave a detailed analysis of this approach in the context of ergodic processes for future research.*

**Remark 12.**  *Several studies, including [49,80,81], have demonstrated that nonlinear wavelet-based thresholding methods often outperform linear approaches when evaluated using the mean integrated error. However, Theorem 3 and its corollary establish that, for any fixed function, g, belonging to the Besov space $B_{s,p,q}$, linear wavelet estimators already achieve the classical optimal convergence rates under the almost-sure sup-norm criterion in the standard i.i.d. framework. This indicates that, in terms of convergence rates, the nonlinear thresholded estimators in (15) do not offer any improvement over their linear counterparts in (2), when β=0 and when the almost-sure sup-norm is used as the evaluation metric.*

*This finding naturally raises the question of why the sup-norm criterion may be preferable to the mean integrated error. A key reason lies in the local behavior of functions within the broad Besov space $B_{s,p,q}$, many of which exhibit pronounced local oscillations. Such fluctuations can lead to significant estimation errors in small regions of $R^{d}$, which may have little impact on integrated error measures but are readily captured by the sup-norm. This issue is particularly relevant for discontinuous functions, where large estimation errors near discontinuities may remain undetected when using the mean integrated error. Empirical evidence from [80,81] supports this argument, highlighting how wavelet-based estimators can exhibit substantial pointwise bias and variance fluctuations that are largely obscured in the mean integrated squared error.*

*Furthermore, in most practical applications, only a single realization of the underlying process is available. The almost-sure sup-norm criterion provides insights into estimator performance for nearly every realization, in contrast to error metrics such as the mean square error or mean integrated squared error, which reflect only the average behavior over multiple realizations. Therefore, we argue that the almost-sure sup-norm serves as a more appropriate benchmark for density and regression estimation in $B_{s,p,q}$. Further discussion on this perspective can be found in [68].*

## 6. Concluding Remarks

This research investigated the estimation of the partial derivatives of multivariate density functions. In particular, we introduced a class of nonparametric estimators based on linear wavelet methods and examined their theoretical properties. We established strong uniform consistency over compact subsets of $R^{d}$ and determined the corresponding rates of convergence. Additionally, we proved the asymptotic normality of the proposed estimators. A significant contribution of this study is the extension of existing results to the setting of ergodic processes.

One of the main open questions in this work concerns the optimal selection of smoothing parameters to minimize the mean squared error (MSE) of the estimators. This remains a crucial problem that warrants further investigation and will be the subject of future research. Another important avenue for exploration involves extending the proposed methodology to functional ergodic data, which presents considerable mathematical challenges beyond the scope of this study. Additionally, a promising research direction is the adaptation of this framework to scenarios with incomplete data, including cases where data are missing at random or subject to censoring under various mechanisms, particularly in the context of spatially dependent data.

An interesting extension of this work would be to relax the assumption of stationarity and consider locally stationary processes, developing comparable theoretical results. However, this generalization would require a fundamentally different mathematical approach, which is left for future study. Another potential research direction involves extending the estimation problem to the framework of stationary continuous time processes, which could provide further insights.

Each of these extensions involves distinct mathematical techniques beyond those employed in the present study and remains an open topic for future exploration. To enhance the practical applicability of the proposed methods, conducting extensive numerical experiments on both simulated and real datasets would be highly valuable, as it would provide empirical support and facilitate more concrete recommendations.

In conclusion, carrying out extensive simulation experiments and analyzing real datasets would greatly enhance the practical value of this study. Such empirical evaluations can provide clear guidelines for effectively implementing these techniques, thereby demonstrating their potential to advance modern statistical analysis.

## 7. Proofs

We derived an upper bound for the partial sums of unbounded martingale differences, which is vital for establishing the asymptotic behavior of the density estimator constructed from strictly stationary and ergodic samples. Throughout the paper, the symbol *C* designates a positive constant whose value may vary from one occurrence to another.

**Proof of Lemma 1. **  The study of the bias term is purely analytical and can be proved by the same arguments as in [27] since it is not affected by the dependence property. Therefore, the details are omitted. □

The relevant upper bound inequality is formulated in the lemmas that follow.

**Lemma 2 (Burkholder–Rosenthal inequality).**  *Following Notation* **1** *in [82], let ${\left(X_{i}\right)}_{i\geq 1}$ be a stationary martingale adapted to the filtration ${\left(F_{i}\right)}_{i\geq 1}$ and define ${\left(d_{i}\right)}_{i\geq 1}$ as the sequence of martingale differences adapted to ${\left(F_{i}\right)}_{i\geq 1}$ and $S_{n}=\sum_{i=1}^{n}d_{i}$; then, for any positive integer, n,*

(16) $$∥\max_{1\leq j\leq n}|S_{j}{|)∥}_{p}\ll n^{1/p}{∥d_{1}∥}_{p}+{∥\sum_{k=1}^{n}E\left(d_{k}^{2}/F_{k-1}\right)∥}_{p/2}^{1/2},\;for\;any\;p\geq 2;$$

*where as usual the norm is ${∥\cdot ∥}_{p}={\left(E[|\cdot {|}^{p}]\right)}^{1/p}$.*

**Lemma 3.**  *Let ${\left(Z_{n}\right)}_{n\geq 1}$ be a sequence of real martingale differences with respect to the sequence of σ-fields ${\left(F_{n}=\sigma \left(Z_{1},\ldots ,Z_{n}\right)\right)}_{n\geq 1}$, where is the σ-field generated by the random variables $Z_{1},\ldots ,Z_{n}$. Set*

$$S_{n}=\sum_{i=1}^{n}Z_{i}.$$

*For any p≥2 and any n≥1, assume that there exist some nonnegative constants, C and $d_{n}$, such that*

$$E\left[Z_{n}^{p}|F_{n-1}\right]\leq C^{p-1}p!\;d_{n}^{2},\;almost\;surely.$$

*Then, for any ϵ>0, we have*

$$P\left(|S_{n}|>\epsilon \right)\leq 2\exp\left\{-\frac{\epsilon^{2}}{2(D_{n}+C\epsilon )}\right\},$$

*where*

$$D_{n}=\sum_{i=1}^{n}d_{i}^{2}.$$

**Proof of Lemma 3. **  The proof follows from a particular case of Theorem 8.2.2 due to [83]. □

In order to establish Theorem 1, we draw upon the following two lemmas.

**Lemma 4.**  *Let $k\in Z^{d}$. Then, under the assumption (C.1), we have*

(17) $$E\left[{\hat{a}}_{m,k}\right]=a_{m,k},\;as\;T\to \infty .$$

**Proof of Lemma 4. **  Consider first the decomposition

(18) $$E\left[{\hat{a}}_{m,k}\right]\;-\;a_{m,k}\;=\;\left(E\left[{\hat{a}}_{m,k}\right]\;-\;{\tilde{a}}_{m,k}\right)\;+\;\left({\tilde{a}}_{m,k}\;-\;a_{m,k}\right)\;=\;A_{m,k,1}\;+\;A_{m,k,2},$$

where

(19) $${\tilde{a}}_{m,k}\;=\;\frac{{\left(-1\right)}^{\left|\beta \right|}}{T}\int_{0}^{T}E\left[\left(\partial^{\beta }\phi_{m,k}\right)\left(X_{i}\right)\;|\;F_{t-\delta }\right]dt.$$

By invoking assumption (C.1), we can further expand ${\tilde{a}}_{m,k}$ as follows:

(20) $$\begin{array}{cc} {\tilde{a}}_{m,k}\; & =\frac{{\left(-1\right)}^{\left|\beta \right|}}{T}\int_{0}^{T}\int_{R^{d}}\left(\partial^{\beta }\phi_{m,k}\right)\left(x\right)f^{F_{t-\delta }}\left(x\right)\;dxdt\\ \; & ={\left(-1\right)}^{\left|\beta \right|}\int_{R^{d}}\left(\partial^{\beta }\phi_{m,k}\right)\left(x\right)\left(\frac{1}{T}\int_{0}^{T}f^{F_{t-\delta }}\left(x\right)dt\right)\;dx\\ \; & ={\left(-1\right)}^{\left|\beta \right|}\int_{R^{d}}\left(\partial^{\beta }\phi_{m,k}\right)\left(x\right)\left(f(x)+o(1)\right)\;dx\\ \; & ={\left(-1\right)}^{\left|\beta \right|}\int_{R^{d}}\left(\partial^{\beta }\phi_{m,k}\right)\left(x\right)f\left(x\right)\;dx\;+\;o\left(1\right)\\ \; & =a_{m,k}\;+\;o\left(1\right). \end{array}$$

Consequently,

(21) $${\tilde{a}}_{m,k}\;=\;a_{m,k}\;\mathrm{as}\;n\;\to \;\infty ,$$

which implies

(22) $$A_{m,k,2}\;=\;o\left(1\right)\;a.s.$$

Hence, we deduce that

$$E\left[{\hat{a}}_{m,k}\right]\;-\;a_{m,k}\;=\;A_{m,k,1}\;+\;o\left(1\right)\;a.s.$$

$A_{m,k,1}$ remains to be analyzed. Notice that by applying assumption (C.1), we have

(23) $$\begin{array}{ccc} A_{m,k,1} & \;=\; & E\left[{\hat{a}}_{m,k}\right]\;-\;{\tilde{a}}_{m,k}\\ & \;=\; & \frac{{\left(-1\right)}^{\left|\beta \right|}}{T}\int_{0}^{T}\left(E\left[\left(\partial^{\beta }\phi_{m,k}\right)\left(X_{t}\right)\right]\;-\;E\left[\left(\partial^{\beta }\phi_{m,k}\right)\left(X_{t}\right)\;|\;F_{t-\delta }\right]dt\right)\\ & \;=\; & \frac{{\left(-1\right)}^{\left|\beta \right|}}{T}\int_{0}^{T}\int_{R^{d}}\left(\partial^{\beta }\phi_{m,k}\right)\left(x\right)\left(f\left(x\right)-f^{F_{t-\delta }}\left(x\right)\right)\;dxdt\\ & = & {\left(-1\right)}^{\left|\beta \right|}\int_{R^{d}}\left(\partial^{\beta }\phi_{m,k}\right)\left(x\right)\left(f\left(x\right)-\left(\frac{1}{T}\int_{0}^{T}f^{F_{t-\delta }}\left(x\right)dt\right)\right)dx\\ & = & o\left(1\right)\left({\left(-1\right)}^{\left|\beta \right|}\int_{R^{d}}\left(\partial^{\beta }\phi_{m,k}\right)\left(x\right)dx\right)\\ & = & o(1), \end{array}$$

since $\left(\partial^{\beta }\phi_{m,k}\right)$ is compactly supported, which implies

(24) $$A_{m,k,1}\;=\;o\left(1\right)\;a.s.$$

The proof of (17) is achieved. □

**Lemma 5.**  *Let $k\in Z^{d}$; then, under the assumptions (C.1) and (C.3), we have*

(25) $$E\left[{\left({\hat{a}}_{m,k}-a_{m,k}\right)}^{2}\right]=O\left(\frac{2^{2m\left(|\beta |-d\right)}}{n}\right),\;as\;T\to \infty .$$

**Proof of Lemma 5. **  Consider the following decomposition:

(26) $${\hat{a}}_{m,k}\;-\;a_{m,k}\;=\;\left({\hat{a}}_{m,k}\;-\;{\tilde{a}}_{m,k}\right)\;+\;\left({\tilde{a}}_{m,k}\;-\;a_{m,k}\right).$$

From this, it follows that

(27) $$\begin{array}{ccc} E\left[{\left({\hat{a}}_{m,k}-a_{m,k}\right)}^{2}\right] & = & E\left[{\left({\hat{a}}_{m,k}-{\tilde{a}}_{m,k}\right)}^{2}\right]\;+\;E\left[{\left({\tilde{a}}_{m,k}-a_{m,k}\right)}^{2}\right]\\ & & \;+\;2\;E\left[\left({\hat{a}}_{m,k}-{\tilde{a}}_{m,k}\right)\left({\tilde{a}}_{m,k}-a_{m,k}\right)\right]\\ & = & A_{m,k,1}\;+\;A_{m,k,2}\;+\;A_{m,k,3}. \end{array}$$

By applying statement (22), one obtains

(28) $$A_{m,k,2}\;=\;o\left(1\right).$$

Moreover, using statements (22) and (24), we deduce

(29) $$\begin{array}{cc} A_{m,k,3}\; & =o\left(1\right)\;E\left[{\hat{a}}_{m,k}\;-\;{\tilde{a}}_{m,k}\right]\\ \; & =o\left(1\right)\;\frac{{\left(-1\right)}^{\left|\beta \right|}}{T}\int_{0}^{T}\left(E\left[\left(\partial^{\beta }\phi_{m,k}\right)\left(X_{t}\right)\right]\;-\;E\left[E\left[\left(\partial^{\beta }\phi_{m,k}\right)\left(X_{t}\right)\;|\;F_{t-\delta }\right]\right]\right)dt\\ \; & =0. \end{array}$$

Consequently, our attention now shifts to the first term in the decomposition (27). Notice that

$$\begin{array}{ccc} {\hat{a}}_{m,k}-{\tilde{a}}_{m,k} & = & \frac{{\left(-1\right)}^{\left|\beta \right|}}{T}\int_{0}^{T}\left(\left(\partial^{\beta }\phi_{m,k}\right)\left(X_{t}\right)\;-\;E\left[\left(\partial^{\beta }\phi_{m,k}\right)\left(X_{t}\right)\;|\;F_{t-\delta }\right]\right)\\ & = & \frac{{\left(-1\right)}^{\left|\beta \right|}}{T}\sum_{i=1}^{n}\Phi_{m,k,i}, \end{array}$$

where

$$\Phi_{m,k,i}=\int_{T_{i-1}}^{T_{i}}\left(\left(\partial^{\beta }\phi_{m,k}\right)\left(X_{t}\right)\;-\;E\left[\left(\partial^{\beta }\phi_{m,k}\right)\left(X_{t}\right)\;|\;F_{t-\delta }\right]\right).$$

Notice that ${\left(\Phi_{m,k,i}\right)}_{0\leq k\leq m_{n}}$ is a sequence of martingale differences with respect to the sequence of σ-fields, ${\left(F_{i-1}\right)}_{0\leq i\leq m_{n}}$. Using the inequality provided by Lemma 2, we immediately deduce

$$A_{m,k,1}\;=\;\frac{{\left(-1\right)}^{2|\beta |}}{T^{2}}\;E\left[{\left|\sum_{i=1}^{n}\Phi_{m,k,i}\right|}^{2}\right].$$

Furthermore, one can establish

(30) $$\begin{array}{ccc} {\left(E\left[{\left|\sum_{i=1}^{n}\Phi_{m,k,i}\right|}^{2}\right]\right)}^{\frac{1}{2}} & \leq & \sqrt{n}\;{∥\Phi_{m,k,1}∥}_{2}\;+\;{∥\sum_{i=1}^{n}E\left[\Phi_{m,k,i}^{2}\;|\;F_{i-2}\right]∥}_{1}^{\frac{1}{2}}\\ & = & \Phi_{\left(1\right)}\;+\;\Phi_{\left(2\right)}. \end{array}$$

To analyze these terms, we use a standard decomposition with Jensen’s inequality and note that $F_{0}$ is the trivial σ-field. Hence, one obtains

(31) 1nΦ(1)2=∥Φm,k,1∥22=E∫0δ∂βϕm,k(Xt)−E∂βϕm,k(Xt)|F0dt2≤∫0δ∑j=022jE∂βϕm,k(Xt)jE∂βϕm,k(Xt)2−jdt=∫0δ∑j=022jE∂βϕm,k(Xt)jE∂βϕm,k(Xt)2−jdt≤4∫0δE∂βϕm,k(Xt)2dt.

Note additionally that

$$\begin{array}{ccc} \int_{0}^{\delta }E\left[{\left|\left(\partial^{\beta }\phi_{m,k}\right)\left(X_{t}\right)\right|}^{2}\right]dt & = & \delta \int_{R^{d}}{\left(\partial^{\beta }\phi_{m,k}\right)}^{2}\left(u\right)\;f\left(u\right)\;du\\ & = & 2^{m\;(d+2|\beta |)}\int_{R^{d}}{\left(\partial^{\beta }\phi \right)}^{2}\left(2^{m}u-k\right)\;f\left(u\right)\;du\\ & = & 2^{2m|\beta |}\int_{R^{d}}{\left(\partial^{\beta }\phi \right)}^{2}\left(v\right)\;f\;\left(\frac{v+k}{2^{m}}\right)\;dv. \end{array}$$

By employing a first-order Taylor expansion alongside Equation (1) and assumption (C.3), we obtain

(32) $$\begin{array}{ccc} \int_{0}^{\delta }E\left[{\left|\left(\partial^{\beta }\phi_{m,k}\right)\left(X_{t}\right)\right|}^{2}\right]dt & = & \delta 2^{2m|\beta |}\int_{R^{d}}{\left(\partial^{\beta }\phi \right)}^{2}\left(v\right)\;\left(f\left(v\right)+O\left(2^{-md}\right)\right)\;dv\\ & = & O\left(2^{m\left(|\beta |\right)}\right). \end{array}$$

This leads to

(33) $$\begin{array}{ccc} \Phi_{1}^{2} & = & 4\left(\delta n\right)2^{2m|\beta |}\int_{R^{d}}{\left(\partial^{\beta }\phi \right)}^{2}\left(v\right)\;\left(f\left(v\right)+O\left(2^{-md}\right)\right)\;dv\\ \Phi_{1} & = & O\left(2^{m\left(|\beta |\right)}T^{1/2}\right). \end{array}$$

Next, we analyze the second component of the decomposition in Equation (30). Specifically, we consider

$$\begin{array}{ccc} \Phi_{2} & = & {\left(E\left(\sum_{i=1}^{n}E\left[\Phi_{m,k,i}^{2}|F_{i-2}\right]\right)\right)}^{1/2}={\left(\sum_{i=1}^{n}E\left[\Phi_{m,k,i}^{2}\right]\right)}^{1/2}. \end{array}$$

Applying a Jensen inequality with a standard identity, we find

$$\begin{array}{ccc} E\left[\Phi_{m,k,i}^{2}\right] & = & E\left[{\left(\int_{T_{i-1}}^{T_{i}}\left(\left(\partial^{\beta }\phi_{m,k}\right)\left(X_{t}\right)\;-\;E\left[\left(\partial^{\beta }\phi_{m,k}\right)\left(X_{t}\right)\;|\;F_{t-\delta }\right]\right)dt\right)}^{2}\right]\\ & \leq & 2\int_{T_{i-1}}^{T_{i}}\left(E\left[|\left(\partial^{\beta }\phi_{m,k}\right)\left(X_{t}\right){|}^{2}\right]dt+E\left[|E\left[\left(\partial^{\beta }\phi_{m,k}\right)\left(X_{t}\right)F_{t-\delta }\right]{|}^{2}\right]\right)dt\\ & \leq & 4\int_{T_{i-1}}^{T_{i}}E\left[|\left(\partial^{\beta }\phi_{m,k}\right)\left(X_{t}\right){|}^{2}\right]dt. \end{array}$$

Using Equation (32), it follows that

(34) $$\begin{array}{ccc} \Phi_{2} & = & O\left(2^{m\left(|\beta |\right)}T^{1/2}\right). \end{array}$$

Combining the results from Equations (33) and (34), we derive

$$\begin{array}{c} {\left(E\left[{\left|\sum_{i=1}^{n}\Phi_{m,k}\left(X_{i}\right)\right|}^{2}\right]\right)}^{\frac{1}{2}}=O\left(2^{m\left(|\beta |\right)}T^{1/2}\right). \end{array}$$

Consequently,

(35) $$\begin{array}{ccc} A_{m,k,1} & = & E\left[{\left({\hat{a}}_{m,k}-{\tilde{a}}_{m,k}\right)}^{2}\right]\\ & = & \frac{{\left(-1\right)}^{2|\beta |}}{T^{2}}\;E\left[{\left|\sum_{i=1}^{n}\Phi_{m,k}\left(X_{i}\right)\right|}^{2}\right]\\ & = & O\left(2^{2m\left(|\beta |\right)}T^{-1}\right). \end{array}$$

Finally, by integrating the findings from Equations (28), (29), and (35), we conclude that

$$E\left[{\left({\hat{a}}_{m,k}-a_{m,k}\right)}^{2}\right]=O\left(\frac{2^{2m\left(|\beta |\right)}}{T}\right).$$

This completes the proof. □

**Proof of Theorem 1.**  The fundamental argument is constructed using established techniques; for a comprehensive exploration, please refer to [24]. Specifically, based on the projected normality’s definition, we define

(36) $$E{∥{\hat{\left(\partial^{\beta }f\right)}}_{T}-\partial^{\beta }f∥}_{2}^{2}=E{∥\sum_{k\in Z^{d}}\left({\hat{a}}_{m,k}-a_{m,k}\right)\phi_{m,k}∥}_{2}^{2}=\sum_{k\in Z^{d}}E\left[{\left({\hat{a}}_{m,k}-a_{m,k}\right)}^{2}\right].$$

According to Lemma 5 and the fact that $|V_{\ell }|∼2^{m}$ and $2^{m}∼n^{\frac{1}{2s+1}}$ and T∼δn,

(37) $$E{∥{\hat{\left(\partial^{\beta }f\right)}}_{T}-\partial^{\beta }f∥}_{2}^{2}=\left(n^{\frac{1}{2s+1}}\right)O\left(\frac{2^{2m\left(|\beta |\right)}}{T}\right)=O\left(T^{-\frac{2(s-|\beta |)}{2s+1}}\right).$$

 □

**Proof of Theorem 2.**  We define

$$L\left(T\right)={\left(\frac{2^{\left(d+2)m(T\right)}T}{\log T}\right)}^{d/2}.$$

Since the domain *D* is compact, it can be covered by a finite number, L=L(T), of cubes, $I_{j}=I_{T,j}$, with centers, $x_{j}=x_{T,j}$, and side lengths, $\ell_{T}$, for j=1,…,L(T). It is straightforward to see that

$$\ell_{n}=\frac{\mathrm{const}}{L^{1/d}\left(T\right)}.$$

Furthermore, we define the term

$${\bar{f}}_{T}\left(x\right)=\frac{{\left(-1\right)}^{\left|\beta \right|}}{Th_{T}^{d+|\beta |}}\int_{0}^{T}E\left[K^{\left(\beta \right)}\left(\frac{x}{h_{T}},\frac{X_{t}}{h_{T}}\right)|F_{t-\delta }\right]dt.$$

Now, we consider the following decomposition:

(38) $$\begin{array}{ccc} \underset{x\in D}{\sup}\left|{\hat{\left(\partial^{\beta }f\right)}}_{T}\left(x\right)-E\left[{\hat{\left(\partial^{\beta }f\right)}}_{T}\left(x\right)\right]\right| & \leq & \underset{x\in D}{\sup}\left|{\hat{\left(\partial^{\beta }f\right)}}_{T}\left(x\right)-{\bar{f}}_{T}\left(x\right)\right|\\ & & +\underset{x\in D}{\sup}\left|{\bar{f}}_{T}\left(x\right)-E\left[{\hat{\left(\partial^{\beta }f\right)}}_{T}\left(x\right)\right]\right|\\ & = & G_{T,1}\left(x\right)+G_{T,2}\left(x\right). \end{array}$$

Making use of the fact that *D* is compact, we readily infer that

(39) $$\begin{array}{ccc} \underset{x\in D}{\sup}\left|{\hat{\left(\partial^{\beta }f\right)}}_{T}\left(x\right)-{\bar{f}}_{T}\left(x\right)\right| & = & \max_{1\leq j\leq L(T)}\underset{x\in D\cap I_{j}}{\sup}\left|{\hat{\left(\partial^{\beta }f\right)}}_{T}\left(x\right)-{\bar{f}}_{T}\left(x\right)\right|\\ & \leq & \max_{1\leq j\leq L(T)}\underset{x\in D\cap I_{j}}{\sup}\left|{\hat{\left(\partial^{\beta }f\right)}}_{T}\left(x\right)-{\hat{\left(\partial^{\beta }f\right)}}_{T}\left(x_{j}\right)\right|\\ & & +\max_{1\leq j\leq L(n)}\left|{\hat{\left(\partial^{\beta }f\right)}}_{T}\left(x_{j}\right)-{\bar{f}}_{T}\left(x_{j}\right)\right|\\ & & +\max_{1\leq j\leq L(T)}\underset{x\in D\cap I_{j}}{\sup}\left|{\bar{f}}_{T}\left(x_{j}\right)-{\bar{f}}_{T}\left(x\right)\right|\\ & = & Q_{1}+Q_{2}+Q_{3}. \end{array}$$

Statement (8) allows us to infer that

$$|{\hat{\left(\partial^{\beta }f\right)}}_{T}\left(x\right)-{\hat{\left(\partial^{\beta }f\right)}}_{T}\left(x_{j}\right)|\leq \frac{d^{1/2}C_{2}}{h_{T}^{d+|\beta |+1}}∥x-x_{j}∥,$$

which implies that

(40) $$Q_{1}\leq \frac{d^{1/2}C_{2}\ell_{T}}{h_{T}^{d+|\beta |+1}}=\frac{const}{L^{1/d}\left(T\right)h_{T}^{d+|\beta |+1}}=O\left({\left(\frac{\log T}{Th_{T}^{d+2|\beta |}}\right)}^{1/2}\right),\;a.s.$$

A similar argument shows, likewise, that

(41) $$Q_{3}\leq \frac{d^{1/2}C_{2}\ell_{T}}{h_{T}^{d+|\beta |+1}}=\frac{const}{L^{1/d}\left(T\right)h_{T}^{d+|\beta |+1}}=O\left({\left(\frac{\log T}{Th_{T}^{d+2|\beta |}}\right)}^{1/2}\right),\;a.s.$$

Now, we turn our attention to the main term and show that

(42) $$Q_{2}=O\left({\left(\frac{\log T}{Th_{T}^{d+2|\beta |}}\right)}^{1/2}\right),\;a.s.$$

Observe that

$$\begin{array}{ccc} Q_{2} & = & \max_{1\leq j\leq L(n)}\left|{\hat{\left(\partial^{\beta }f\right)}}_{T}\left(x_{j}\right)-{\bar{f}}_{T}\left(x_{j}\right)\right|\\ & = & \max_{1\leq j\leq L(T)}\left|\frac{{\left(-1\right)}^{\left|\beta \right|}}{Th_{T}^{d+|\beta |}}\sum_{i=1}^{n}\int_{T_{i-1}}^{T_{i}}\left(K^{\left(\beta \right)}\left(\frac{x_{j}}{h_{T}},\frac{X_{t}}{h_{T}}\right)-E\left[K^{\left(\beta \right)}\left(\frac{x_{j}}{h_{T}},\frac{X_{t}}{h_{T}}\right)|F_{t-\delta }\right]\right)dt\right|\\ & = & \max_{1\leq j\leq L(T)}\left|\frac{{\left(-1\right)}^{\left|\beta \right|}}{Th_{T}^{d+|\beta |}}\sum_{i=1}^{n}Z_{n,i}\left(x_{j}\right)\right|, \end{array}$$

where, for i=1,…,n,

$$Z_{n,i}\left(x_{j}\right)={\left(-1\right)}^{\left|\beta \right|}\int_{T_{i-1}}^{T_{i}}\left(K^{\left(\beta \right)}\left(\frac{x_{j}}{h_{T}},\frac{X_{t}}{h_{T}}\right)-E\left[K^{\left(\beta \right)}\left(\frac{x_{j}}{h_{T}},\frac{X_{t}}{h_{T}}\right)|F_{t-\delta }\right]\right)dt,$$

is a sequence of martingale difference arrays with respect to the σ-field $F_{i-1}$. Observe that by applying the Jensen then Minkowski inequalities, we obtain

(43) $$\begin{array}{c} \left|E\left[{\left(Z_{n,i}\left(x_{j}\right)\right)}^{p}|F_{i-2}\right]\right|\\ \;\leq \int_{T_{i-1}}^{T_{i}}E\left[{\left|K^{\left(\beta \right)}\left(\frac{x_{j}}{h_{T}},\frac{X_{t}}{h_{T}}\right)-E\left[K^{\left(\beta \right)}\left(\frac{x_{j}}{h_{T}},\frac{X_{t}}{h_{T}}\right)|F_{t-\delta }\right]\right|}^{p}|F_{i-2}\right]dt\\ \;=\int_{T_{i-1}}^{T_{i}}\left(E^{1/p}\left[{\left(K^{\left(\beta \right)}\right)}^{p}\left(\frac{x_{j}}{h_{T}},\frac{X_{t}}{h_{T}}\right)|F_{i-2}\right]\right.\\ \;\;\left.+E^{1/p}\left[{\left(E\left[K^{\left(\beta \right)}\left(\frac{x_{j}}{h_{T}},\frac{X_{t}}{h_{T}}\right)|F_{t-\delta }\right]\right)}^{p}|F_{i-2}\right]\right)dt. \end{array}$$

By using the fact that ${\left(E\left[K^{\left(\beta \right)}\left(\frac{x_{j}}{h_{T}},\frac{X_{t}}{h_{T}}\right)|F_{t-\delta }\right]\right)}^{p-k}$ is $F_{i-2}$-measurable since all $\delta \in [T_{i-1},T_{i})$, we have

$$F_{i-2}\subset F_{t-\delta },$$

It follows from (43) that

$$\begin{array}{ccc} \left|E\left[{\left(Z_{n,i}\left(x_{j}\right)\right)}^{p}|F_{i-2}\right]\right| & \leq & 2^{p}\int_{T_{i-1}}^{T_{i}}E^{1/p}\left[{\left(K^{\left(\beta \right)}\right)}^{p}\left(\frac{x_{j}}{h_{T}},\frac{X_{t}}{h_{T}}\right)|F_{i-2}\right]dt. \end{array}$$

It follows readily from (6), for any integer, ζ, that

(44) $$\begin{array}{c} E\left[{\left(K^{\left(\beta \right)}\right)}^{p}\left(\frac{x_{j}}{h_{T}},\frac{X_{t}}{h_{T}}\right)|F_{i-2}\right]\\ \;=\int_{R^{d}}{\left(K^{\left(\beta \right)}\right)}^{p}\left(\frac{x_{j}}{h_{T}},\frac{y}{h_{T}}\right)f^{F_{i-2}}\left(y\right)dy\\ \;\leq \int_{R^{d}}{\left(\frac{C_{d+|\beta |}}{(1+h_{n}^{-1}∥x_{j}-y{{∥}_{2})}^{d+|\beta |}}\right)}^{p}f^{F_{i-2}}\left(y\right)dy\\ \;=\int_{R^{d}}{\left(\frac{h_{T}^{d+|\beta |}C_{d+|\beta |}}{(h_{T}+∥x_{j}-y{∥)}^{d+|\beta |}}\right)}^{p}f^{F_{i-2}}\left(y\right)dy\\ \;\leq h_{T}^{p(d+|\beta |)}C_{d+|\beta |}^{p}\int_{R^{d}}f^{F_{i-2}}\left(y\right)dy=h_{T}^{p(d+|\beta |)}C_{d+|\beta |}^{p}, \end{array}$$

which gives the following upper bound:

$$\begin{array}{ccc} \left|E\left[{\left(Z_{n,i}\left(x_{j}\right)\right)}^{p}|F_{i-2}\right]\right| & \leq & 2^{p}\delta h_{T}^{p(d+|\beta |)}C_{d+|\beta |}^{p}\leq p!C^{p-1}d_{i}^{2}. \end{array}$$

We shall apply Lemma 3 to the sum of $\left\{Z_{n}\left(x_{j},X_{i}\right)\right\}$, with

$$C=2C_{d+|\beta |},\;\;d_{i}^{2}=C_{d+|\beta |}\delta h_{T}^{d+|\beta |},\;\;D_{n}=\sum_{i=1}^{n}d_{i}^{2}=O\left(Th_{T}^{d+|\beta |}\right)$$

and

$$\epsilon_{T}=\epsilon_{0}{\left(\log T/Th_{T}^{d+2|\beta |}\right)}^{1/2}.$$

Therefore, we have the following chain of inequalities for some positive constant, $C_{2}$:

$$\begin{array}{c} P\left(\max_{1\leq j\leq L(T)}\left|\frac{1}{Th_{T}^{d+|\beta |}}\sum_{i=1}^{n}Z_{n,i}\left(x_{j}\right)\right|>\epsilon_{T}\right)\\ \;\leq \sum_{j=1}^{L(T)}P\left(\left|\sum_{i=1}^{n}Z_{n,i}\left(x_{j}\right)\right|>\epsilon_{T}Th_{T}^{d+|\beta |}\right)\\ \;\leq 2L\left(T\right)\exp\left\{-\frac{\epsilon_{0}^{2}{\left(Th_{T}^{d+|\beta |}\right)}^{2}\left(\log T/Th_{T}^{d+2|\beta |}\right)}{2(D_{n}+2C_{d+1}Th_{T}^{d+|\beta |}{\left(\log T/Th_{T}^{d+2|\beta |}\right)}^{1/2})}\right\}\\ \;\leq 2{\left(\frac{T}{h_{T}^{d+1}\log T}\right)}^{d/2}\exp\left\{-\frac{\epsilon_{0}^{2}Th_{T}^{d+|\beta |}\left(\log T/h_{T}^{\left|\beta \right|}\right)}{O\left(Th_{T}^{d+|\beta |}\right)\left(1+2C_{d+1}{\left(\log T/Th_{T}^{d+2|\beta |}\right)}^{1/2}\right)}\right\}\\ \;\leq 2{\left(\frac{T}{h_{T}^{d+1}\log T}\right)}^{d/2}\left(T^{-\frac{C_{2}\epsilon_{0}^{2}}{h_{T}^{\left|\beta \right|}}}\right)\\ \;=\frac{2T^{d/2-\frac{C_{2}\epsilon_{0}^{2}}{h_{T}^{\left|\beta \right|}}}}{{\left(h_{T}^{d+1}\log T\right)}^{d/2}}\\ \;=\frac{2}{{\left((Th_{T}^{d+1})\log T\right)}^{d/2}T^{(C_{2}\epsilon_{0}^{2}/h_{T}^{\left|\beta \right|})-d}}. \end{array}$$

Observe that

$$\frac{1}{h_{T}^{\left|\beta \right|}}\to \infty \;\mathrm{as}\;n\to \infty .$$

We choose a sufficiently large $\epsilon_{0}$, such that

$$(C_{2}\epsilon_{0}^{2}/h_{T}^{\left|\beta \right|})-d>0.$$

We readily obtain that

(45) $$\sum_{n\geq 1}P\left(\max_{1\leq j\leq L(T)}\left|\frac{1}{Th_{T}^{d+|\beta |}}\sum_{i=1}^{n}Z_{n,i}\left(x_{j}\right)\right|>\epsilon_{T}\right)<\infty .$$

We obtain assertion (42) through a routine application of the Borel–Cantelli lemma. Therefore, the conclusion of Theorem 2 follows, using decomposition (39) in combination with (40)–(42). The second term on the right side of (38) remains to be evaluated. One can see that

$$\begin{array}{c} \underset{x\in D}{\sup}\left|{\bar{f}}_{T}\left(x\right)-E\left[{\hat{\left(\partial^{\beta }f\right)}}_{T}\left(x\right)\right]\right|\\ \;=\underset{x\in D}{\sup}\left|\frac{{\left(-1\right)}^{\left|\beta \right|}}{Th_{T}^{d+|\beta |}}\int_{0}^{T}\left(E\left[K^{\beta }\left(\frac{x}{h_{T}},\frac{X_{t}}{h_{T}}\right)|F_{t-\delta }\right]-E\left[K^{\beta }\left(\frac{x}{h_{T}},\frac{X_{t}}{h_{T}}\right)\right]\right)dt\right|\\ \;=\underset{x\in D}{\sup}\left|\frac{{\left(-1\right)}^{\left|\beta \right|}}{Th_{T}^{d+|\beta |}}\int_{0}^{T}\int_{R^{d}}K^{\beta }\left(\frac{x}{h_{T}},\frac{y}{h_{T}}\right)\left(f^{F_{t-\delta }}\left(y\right)-f\left(y\right)\right)dydt\right|\\ \;=\underset{x\in D}{\sup}\left|\frac{{\left(-1\right)}^{\left|\beta \right|}}{h_{T}^{d+|\beta |}}\int_{R^{d}}K^{\beta }\left(\frac{x}{h_{T}},\frac{y}{h_{T}}\right)\left(\frac{1}{T}\int_{0}^{T}f^{F_{t-\delta }}\left(y\right)dt-f\left(y\right)\right)dy\right|. \end{array}$$

Making use of the Cauchy–Schwarz inequality and statement (6) when m=d+2|β|+1, we readily obtain that

$$\begin{array}{c} \left|\frac{{\left(-1\right)}^{\left|\beta \right|}}{h_{T}^{d+|\beta |}}\int_{R^{d}}K^{\beta }\left(\frac{x}{h_{T}},\frac{y}{h_{T}}\right)\left(\frac{1}{T}\int_{0}^{T}f^{F_{t-\delta }}\left(y\right)dt-f\left(y\right)\right)dy\right|\\ \;\leq {\left(\int_{R^{d}}{\left|\frac{1}{h_{n}^{d+|\beta |}}K^{\beta }\left(\frac{x}{h_{n}},\frac{y}{h_{n}}\right)\right|}^{2}dy\right)}^{1/2}{\left(\int_{R^{d}}|\frac{1}{T}\int_{0}^{T}f^{F_{t-\delta }}\left(y\right)dt-f\left(y\right){|}^{2}dy\right)}^{1/2}\\ \;\leq ∥\frac{1}{T}\int_{0}^{T}f^{F_{t-\delta }}dt-f{∥}_{L^{2}}{\left(\int_{R^{d}}\left(\frac{1}{h_{T}^{d+2|\beta |}}\frac{C_{d+2|\beta |+1}}{(1+h_{T}^{-1}{∥x-y∥)}^{d+2|\beta |+1}}\right)\left|\frac{1}{h_{T}^{d}}K^{\beta }\left(\frac{x}{h_{T}},\frac{y}{h_{T}}\right)\right|dy\right)}^{1/2}\\ \;\leq h_{T}^{1/2}C_{d+2|\beta |+1}∥\frac{1}{T}\int_{0}^{T}f^{F_{t-\delta }}dt-f{∥}_{L^{2}}G_{1}^{1/2}\left(d\right). \end{array}$$

Under assumption (C.2), we deduce that

(46) $$\begin{array}{ccc} G_{n,2}\left(x\right) & = & \underset{x\in D}{\sup}\left|{\bar{f}}_{n}\left(x\right)-E\left[{\hat{\left(\partial^{\beta }f\right)}}_{n}\left(x\right)\right]\right|\leq h_{T}^{1/2}C_{d+2|\beta |+1}G_{1}^{1/2}\left(d\right)\\ & = & O(h_{T}^{1/2}). \end{array}$$

Hence, the proof is completed. □

**Proof of Theorem 3.**  Recall the decomposition

(47) $$\begin{array}{c} \sqrt{Th_{T}^{d+2|\beta |}}\left({\hat{\left(\partial^{\beta }f\right)}}_{T}\left(x\right)-\left(\partial^{\beta }f\right)\left(x\right)\right)\\ \;=\sqrt{Th_{T}^{d+2|\beta |}}\left(\left({\hat{\left(\partial^{\beta }f\right)}}_{n}\left(x\right)-{\bar{f}}_{T}\left(x\right)\right)+\left({\bar{f}}_{T}\left(x\right)-\hat{\left(\partial^{\beta }f\right)}\left(x\right)\right)\right)\\ \;=\sqrt{Th_{T}^{d+2|\beta |}}\left(Q_{T}\left(x\right)+B_{T}\left(x\right)\right). \end{array}$$

Observe that

(48) $$\begin{array}{ccc} B_{T}\left(x\right) & = & \left({\bar{f}}_{T}\left(x\right)-E\left[{\hat{\left(\partial^{\beta }f\right)}}_{T}\left(x\right)\right]\right)+\left(E\left[{\hat{\left(\partial^{\beta }f\right)}}_{T}\left(x\right)\right]-\hat{\left(\partial^{\beta }f\right)}\left(x\right)\right)\\ & = & B_{T,1}\left(x\right)+B_{T,2}\left(x\right). \end{array}$$

Hence, using a similar argument as for (46) and under hypothesis (C.2) and statement (6) when m=2(d+|β|) and condition (10), we readily obtain that

(49) $$\begin{array}{c} {\left(Th_{T}^{d+2|\beta |}\right)}^{1/2}B_{T,1}\left(x\right)\\ \;={\left(Th_{T}^{d+2|\beta |}\right)}^{1/2}\left(\frac{{\left(-1\right)}^{\left|\beta \right|}}{Th_{T}^{d+|\beta |}}\int_{0}^{T}\left(E\left[K^{\beta }\left(\frac{x}{h_{T}},\frac{X_{t}}{h_{T}}\right)|F_{t-\delta }\right]-E\left[K^{\beta }\left(\frac{x}{h_{T}},\frac{X_{t}}{h_{T}}\right)\right]\right)dt\right)\\ \;=O(h_{T}^{d/2}{\left(Th_{T}^{d+2|\beta |}\right)}^{1/2}=o\left(1\right). \end{array}$$

On the other hand, according to condition (10) and Lemma 1, we have

(50) $${\left(Th_{T}^{d+2|\beta |}\right)}^{1/2}B_{T,2}\left(x\right)=O\left(h_{T}^{\delta }{\left(Th_{T}^{d+2|\beta }\right)}^{1/2}\right)=o\left(1\right).$$

Observe that

(51) $$\begin{array}{c} \sqrt{Th_{T}^{d+2|\beta |}}Q_{T}\left(x\right)\\ \;=\sum_{i=1}^{n}\left\{\int_{T_{i-1}}^{T_{i}}\left(\frac{{\left(-1\right)}^{\left|\beta \right|}}{\sqrt{Th_{T}^{d}}}K^{\left(\beta \right)}\left(\frac{x}{h_{T}},\frac{X_{t}}{h_{T}}\right)\right)dt-\int_{T_{i-1}}^{T_{i}}E\left[\frac{{\left(-1\right)}^{\left|\beta \right|}}{\sqrt{Th_{T}^{d}}}K^{\left(\beta \right)}\left(\frac{x}{h_{T}},\frac{X_{t}}{h_{T}}\right)|F_{t-\delta }\right]dt\right\}\\ \;=\sum_{i=1}^{n}\left\{\xi_{ni}\left(x\right)-{\bar{\xi }}_{ni}\left(x\right)\right\}\\ \;=\sum_{i=1}^{n}\chi_{ni}\left(x\right), \end{array}$$

where

$$\begin{array}{ccc} \chi_{ni}\left(x\right) & = & \xi_{ni}\left(x\right)-{\bar{\xi }}_{ni}\left(x\right),\\ \xi_{ni}\left(x\right) & = & \frac{{\left(-1\right)}^{\left|\beta \right|}}{\sqrt{Th_{T}^{d}}}\int_{T_{i-1}}^{T_{i}}\left(K^{\left(\beta \right)}\left(\frac{x}{h_{T}},\frac{X_{t}}{h_{T}}\right)\right)dt,\\ {\bar{\xi }}_{ni}\left(x\right) & = & \frac{{\left(-1\right)}^{\left|\beta \right|}}{\sqrt{Th_{T}^{d}}}\int_{T_{i-1}}^{T_{i}}E\left[K^{\left(\beta \right)}\left(\frac{x}{h_{T}},\frac{X_{t}}{h_{T}}\right)|F_{t-\delta }\right]dt, \end{array}$$

where $\chi_{ni}\left(x,\varphi \right)$ is a triangular array of martingale differences with respect to the σ-field $F_{i-1}$. This allows us to apply the central limit theorem for discrete time arrays of martingales (see [84]) to establish the asymptotic normality of $\sqrt{nh_{n}^{d+2|\beta |}}Q_{n}\left(x\right)$. This can be performed if we establish the following statements:

(a) Lyapunov condition:
  $$\sum_{i=1}^{n}E\left[\chi_{ni}^{2}\left(x\right)|F_{i-2}\right]\overset{P}{\to }\Sigma^{2}\left(x\right);$$
(b) Lindberg condition:
  $$nE\left[\chi_{ni}^{2}\left(x\right)1_{\left\{\left|\chi_{ni}\left(x\right)\right|>\epsilon \right\}}\right]=o\left(1\right),\;\;\mathrm{holds}\;\mathrm{true}\;\mathrm{for}\;\mathrm{any}\;\;\epsilon >0.$$

**Proof of part (a)**  Observe that

$$\left|\sum_{i=1}^{n}E\left[\xi_{ni}^{2}\left(x_{i}\right)|F_{i-2}\right]-\sum_{i=1}^{n}E\left[\chi_{ni}^{2}\left(x\right)|F_{i-2}\right]\right|=\sum_{i=1}^{n}{\left(E\left[\xi_{ni}\left(x\right)|F_{i-2}\right]\right)}^{2}.$$

By employing a first-order Taylor expansion alongside Equation (6) and assumption (C.4), we obtain

$$\begin{array}{ccc} E\left[\xi_{ni}\left(x\right)|F_{i-2}\right] & = & \frac{{\left(-1\right)}^{\left|\beta \right|}}{\sqrt{nh_{n}^{d}}}\int_{T_{i-1}}^{T_{i}}E\left[\left(K^{\left(\beta \right)}\left(\frac{x}{h_{T}},\frac{X_{t}}{h_{T}}\right)\right)|F_{i-2}\right]dt\\ & = & \frac{{\left(-1\right)}^{\left|\beta \right|}}{\sqrt{nh_{n}^{d}}}\int_{T_{i-1}}^{T_{i}}\int_{R^{d}}K^{\beta }\left(\frac{x}{h_{n}},\frac{y}{h_{n}}\right)f_{t}^{F_{i-2}}\left(y\right)dydt\\ & = & \frac{{\left(-1\right)}^{\left|\beta \right|}\sqrt{h_{n}^{d}}}{\sqrt{n}}\int_{T_{i-1}}^{T_{i}}\int_{R^{d}}K^{\beta }\left(\frac{x}{h_{n}},\frac{x}{h_{n}}+u\right)f_{t}^{F_{i-2}}\left(x+h_{n}u\right)dudt\\ & \leq & \left(\frac{{\left(-1\right)}^{\left|\beta \right|}\sqrt{h_{n}^{d}}}{\sqrt{n}}\right)\left(\frac{C_{d+|\beta |}}{{(1+∥u∥}_{2}{)}^{d+|\beta |}}\right)\left(\int_{T_{i-1}}^{T_{i}}f_{t}^{F_{i-2}}\left(x\right)dt+o\left(1\right)\right). \end{array}$$

Let

$$g_{i-1}^{F_{i-2}}\left(x\right)={\left(\int_{T_{i-1}}^{T_{i}}f_{t}^{F_{i-2}}\left(x\right)dt\right)}^{2}.$$

Making use of the Riemann sum, the quantity $g_{i-1}^{F_{i-2}}\left(x\right)$ may be approached, whenever δ is small enough, by $\delta f_{T_{i-1}}^{F_{i-2}}$. It is then clear from the discussion above that the process ${\left(g_{T_{i-1}}^{F_{i-2}}\right)}_{i\geq 1}$ is stationary and ergodic. So, the sum $\frac{1}{n}\sum_{i=1}^{n}g_{i-1}^{F_{i-2}}\left(x\right)$ has a finite limit, (see [85], Theorem 4.4), which is

(52) $$E\left[f_{0}^{F_{-\delta }}\left(x\right)\right]={\left(\int_{0}^{\delta }f\left(x\right)dt\right)}^{2}=\delta^{2}f^{2}\left(x\right),$$

where $F_{-\delta }$ is the trivial σ-field. Therefore,

$$\begin{array}{c} \sum_{i=1}^{n}{\left(E\left[\xi_{ni}\left(x\right)|F_{i-2}\right]\right)}^{2}\\ \;=\left(h_{n}^{d}\right){\left(\frac{C_{d+|\beta |}}{{(1+∥u∥}_{2}{)}^{d+|\beta |}}\right)}^{2}\left(\frac{1}{n}\sum_{i=1}^{n}{\left(\int_{T_{i-1}}^{T_{i}}f_{t}^{F_{i-2}}\left(x\right)dt\right)}^{2}+o\left(1\right)\right)=O\left(h_{n}^{d}\right). \end{array}$$

Statement (a) then follows from

$$\lim_{n\to \infty }\sum_{i=1}^{n}E\left[\xi_{ni}^{2}\left(x\right)|G_{i-1}\right]\overset{P}{=}\Sigma_{\varphi }^{2}\left(x\right).$$

Observe the fact that (see [42])

$$K^{\left(\beta \right)}\left(u,v\right)=K^{\left(\beta \right)}\left(u+k,v+k\right),\;\;for\;\;k\in Z^{d}.$$

According to assumptions (C.1) and (C.4), we have

$$\begin{array}{c} \sum_{i=1}^{n}E\left[\xi_{ni}^{2}\left(x\right)|F_{i-2}\right]\\ \;=\sum_{i=1}^{n}E\left[{\left(\frac{{\left(-1\right)}^{\left|\beta \right|}}{\sqrt{Th_{T}^{d}}}\int_{T_{i-1}}^{T_{i}}K^{\left(\beta \right)}\left(\frac{x}{h_{T}},\frac{X_{t}}{h_{T}}\right)dt\right)}^{2}|F_{i-1}\right]\\ \;\leq \frac{1}{Th_{T}^{d}}\sum_{i=1}^{n}\int_{T_{i-1}}^{T_{i}}\int_{R^{d}}{\left(K^{\left(\beta \right)}\right)}^{2}\left(\frac{x}{h_{T}},\frac{u}{h_{T}}\right)f_{t}^{F_{i-2}}\left(u\right)dudt\\ \;=\frac{1}{n\delta }\sum_{i=1}^{n}\int_{T_{i-1}}^{T_{i}}\int_{R^{d}}{\left(K^{\left(\beta \right)}\right)}^{2}\left(0,v\right)f_{t}^{F_{i-2}}\left(x+h_{T}v\right)dvdt\\ \;=\frac{1}{\delta }\left(\int_{R^{d}}{\left(K^{\left(\beta \right)}\right)}^{2}\left(0,v\right)dv\right)\left(\frac{1}{n}\sum_{i=1}^{n}\left(\int_{T_{i-1}}^{T_{i}}f_{t}^{F_{i-2}}\left(x\right)dt\right)+o\left(1\right)\right)\\ \;=\frac{1}{\delta }\left(\delta f(x)+o(1)\right)\left(\int_{R^{d}}{\left(K^{\left(\beta \right)}\right)}^{2}\left(0,v\right)dv\right). \end{array}$$

We deduce

(53) $$\lim_{n\to \infty }\sum_{i=1}^{n}E\left[\xi_{ni}^{2}\left(x\right)|F_{i-2}\right]=f\left(x\right)\int_{R^{d}}{\left(K^{\left(\beta \right)}\right)}^{2}\left(0,v\right)dv.$$

 □

**Proof of part (b).**  The Lindberg condition results from Corollary 9.5.2 in [86], which implies that

$$nE\left[\chi_{ni}^{2}\left(x\right)1_{\left\{\left|\chi_{ni}\left(x\right)\right|>\epsilon \right\}}\right]\leq 4nE\left[\xi_{ni}^{2}\left(x\right)1_{\left\{\left|\xi_{ni}\left(x\right)\right|>\epsilon /2\right\}}\right].$$

Let a>1 and b>1 such that

$$\frac{1}{a}+\frac{1}{b}=1.$$

Making use of Hölder and Markov inequalities, one can write, for all ϵ>0,

$$\begin{array}{ccc} E\left[\xi_{ni}^{2}\left(x\right)1_{\left\{\left|\xi_{ni}\left(x\right)\right|>\epsilon /2\right\}}\right] & \leq & \frac{E{\left|\xi_{ni}\left(x\right)\right|}^{2a}}{{\left(\epsilon /2\right)}^{2a/b}}. \end{array}$$

Therefore, by using condition (6) and assumption (C.3), we obtain

$$\begin{array}{c} 4nE\left[\xi_{ni}^{2}\left(x\right)1_{\left\{\left|\xi_{ni}\left(x\right)\right|>\epsilon /2\right\}}\right]\\ \;\leq \frac{4}{T^{a-1}h_{T}^{ad}{\left(\epsilon /2\right)}^{2a/b}}\int_{T_{i-1}}^{T_{i}}E{\left|\left(K^{\left(\beta \right)}\left(\frac{x}{h_{T}},\frac{X_{t}}{h_{T}}\right)\right)\right|}^{2a}dt\\ \;=\frac{4}{T^{a-1}h_{n}^{ad}{\left(\epsilon /2\right)}^{2a/b}}\int_{T_{i-1}}^{T_{i}}\int_{R^{d}}{\left(K^{\left(\beta \right)}\right)}^{2a}\left(\frac{x}{h_{T}},\frac{u}{h_{T}}\right)f\left(u\right)dudt\\ \;=\frac{4}{T^{a-1}h_{T}^{(a-1)d}{\left(\epsilon /2\right)}^{2a/b}}\int_{T_{i-1}}^{T_{i}}\int_{R^{d}}{\left(K^{\left(\beta \right)}\right)}^{2a}\left(0,v\right)f\left(x+h_{T}v\right)dvdt\\ \;=\frac{4}{T^{a-1}h_{T}^{(a-1)d}{\left(\epsilon /2\right)}^{2a/b}}\int_{T_{i-1}}^{T_{i}}\int_{R^{d}}{\left(K^{\left(\beta \right)}\right)}^{2a}\left(0,v\right)\left(f(x)+o(1)\right)dvdt. \end{array}$$

We infer that

(54) $$\begin{array}{ccc} 4nE\left[\xi_{ni}^{2}\left(x,\varphi \right)1_{\left\{\left|\xi_{ni}\left(x,\varphi \right)\right|>\epsilon /2\right\}}\right] & \leq & \frac{4C_{d+1}^{2a}}{T^{a-1}h_{T}^{(a-1)d}{\left(\epsilon /2\right)}^{2a/b}}\\ & = & O\left({\left(\frac{1}{Th_{T}^{d}}\right)}^{a-1}\right). \end{array}$$

Combining statements (53) and (54), we achieve the proof of the theorem. □

 □

## Data Availability

Data are contained within the article.

## Appendix A. Besov Spaces

There are many equivalent definitions of Besov spaces; see, e.g., [87]. Following [68], consider the parameters 1≤p,q≤∞, and define the shift operator $S_{\tau }$ acting on a function, *f*, as

$$\left(S_{\tau }f\right)\left(x\right)=f\left(x-\tau \right).$$

For a fractional order, 0<s<1, introduce the seminorm

$$\gamma_{s,p,q}\left(f\right)={\left(\int_{R^{d}}{\left(\frac{∥S_{\tau }{f-f∥}_{L_{p}}}{{∥\tau ∥}^{s}}\right)}^{q}\frac{d\tau }{{∥\tau ∥}^{d}}\right)}^{1/q},$$

with the limiting case when q=∞, given by

$$\gamma_{s,p,\infty }\left(f\right)=\underset{\tau \in R^{d}}{\sup}\frac{∥S_{\tau }{f-f∥}_{L_{p}}}{{∥\tau ∥}^{s}}.$$

For the special case s=1, the seminorm takes the form

$$\gamma_{1,p,q}\left(f\right)={\left(\int_{R^{d}}{\left(\frac{∥S_{\tau }f+S_{-\tau }{f-2f∥}_{L_{p}}}{∥\tau ∥}\right)}^{q}\frac{d\tau }{{∥\tau ∥}^{d}}\right)}^{1/q},$$

and in the supremum norm,

$$\gamma_{1,p,\infty }\left(f\right)=\underset{\tau \in R^{d}}{\sup}\frac{∥S_{\tau }f+S_{-\tau }{f-2f∥}_{L_{p}}}{∥\tau ∥}.$$

The Besov space $B_{s,p,q}$ for 0<s<1 and 1≤p,q≤∞ consists of all functions, $f\in L_{p}\left(R^{d}\right)$, for which $\gamma_{s,p,q}\left(f\right)$ is finite:

$$B_{s,p,q}=\left\{f\in L_{p}\left(R^{d}\right):\gamma_{s,p,q}\left(f\right)<\infty \right\}.$$

For higher regularity levels, where s>1, decompose *s* into its integer and fractional components as $s={\left[s\right]}^{-}+{\left\{s\right\}}^{+}$, where ${\left[s\right]}^{-}$ is the greatest integer less than or equal to *s* and $0<{\left\{s\right\}}^{+}\leq 1$. In this case, the Besov space $B_{s,p,q}$ comprises functions, $f\in L_{p}\left(R^{d}\right)$, whose weak derivatives, $D^{j}f$, belong to $B_{{\left\{s\right\}}^{+},p,q}$ for all multi-indices, *j*, satisfying $\left|j\right|\leq {\left[s\right]}^{-}$. The associated norm is given by

$${∥f∥}_{B_{s,p,q}}={∥f∥}_{L_{p}}+\sum_{\left|j\right|\leq {\left[s\right]}^{-}}\gamma_{{\left\{s\right\}}^{+},p,q}\left(D^{j}f\right).$$

classically differentiable according to the fundamental theorem of calculus. One defines the Sobolev spaces of weakly differentiable functions on *A*; for 1≤p<∞, the $L^{p}$ Sobolev space of the order s∈N is defined as

$$H_{p}^{s}\left(A\right)=\left\{f\in L^{p}:\partial^{j}f\in L^{p}\left(A\right),\forall j={1,\ldots ,s:∥f∥}_{H_{p}^{s}\left(A\right)}\equiv {∥f∥}_{p}+{∥\partial^{s}f∥}_{p}<\infty \right\},$$

Notable instances of Besov spaces include the Sobolev space $H_{2}^{s}=B_{s,2,2}$ and the space of bounded *s*-Lipschitz functions, $B_{s,\infty ,\infty }$.

**Remark A1.** 
*It is worth noting that the optimal $L^{2}$ risk for density estimation on a Sobolev ball with the regularity index s is of the order $O\left(n^{-2s/(2s+1)}\right)$; see [88,89,90]. For a comprehensive examination of the connections between classical function spaces and Besov spaces—including the Fourier analytical characterizations of Sobolev spaces for p≠2—the reader is referred to [91,92]. In ref. [93], the relationship between $V_{p}$ spaces (comprising functions of bounded p-variation) and Besov spaces is explored further, drawing on interpolation methods detailed in [52]. An alternative, more traditional treatment of p-variation spaces can be found in [94]. Moreover, an important reference addressing Besov spaces defined on broader geometric structures, such as manifolds and Dirichlet spaces, is [95].*
